# Functional Features and Current Applications of the RNA‐Targeting Type VI CRISPR‐Cas Systems

**DOI:** 10.1002/advs.202004685

**Published:** 2021-05-05

**Authors:** Vanja Perčulija, Jinying Lin, Bo Zhang, Songying Ouyang

**Affiliations:** ^1^ The Key Laboratory of Innate Immune Biology of Fujian Province Provincial University Key Laboratory of Cellular Stress Response and Metabolic Regulation Biomedical Research Center of South China Key Laboratory of OptoElectronic Science and Technology for Medicine of the Ministry of Education College of Life Sciences Fujian Normal University Fuzhou 350117 China; ^2^ International College of Chinese Studies Fujian Normal University Fuzhou 350117 China; ^3^ Laboratory for Marine Biology and Biotechnology Pilot National Laboratory for Marine Science and Technology (Qingdao) Qingdao 266237 China; ^4^ National Laboratory of Biomacromolecules Institute of Biophysics Chinese Academy of Sciences Beijing 100101 China

**Keywords:** Cas13, CRISPR‐Cas, RNA interference, transcriptome engineering, type VI CRISPR‐Cas systems

## Abstract

CRISPR‐Cas systems are a form of prokaryotic adaptive immunity that employs RNA‐guided endonucleases (Cas effectors) to cleave foreign genetic elements. Due to their simplicity, targeting programmability, and efficiency, single‐effector CRISPR‐Cas systems have great potential for application in research, biotechnology, and therapeutics. While DNA‐targeting Cas effectors such as Cas9 and Cas12a have become indispensable tools for genome editing in the past decade, the more recent discovery of RNA‐targeting CRISPR‐Cas systems has opened the door for implementation of CRISPR‐Cas technology in RNA manipulation. With an increasing number of studies reporting their application in transcriptome engineering, viral interference, nucleic acid detection, and RNA imaging, type VI CRISPR‐Cas systems and the associated Cas13 effectors particularly hold promise as RNA‐targeting or RNA‐binding tools. However, even though previous structural and biochemical characterization provided a firm basis for leveraging type VI CRISPR‐Cas systems into such tools, the lack of comprehension of certain mechanisms underlying their functions hinders more sophisticated and conventional use. This review will summarize current knowledge on structural and mechanistic properties of type VI CRISPR‐Cas systems, give an overview on the reported applications, and discuss functional features that need further investigation in order to improve performance of Cas13‐based tools.

## Graphical abstract

Class 2 type VI CRISPR‐Cas systems are characterized by Cas13 effector proteins that exclusively cleave single‐stranded RNA. This review article summarizes current knowledge on structural and mechanistic properties of type VI CRISPR‐Cas systems, gives an overview on reported applications of Cas13‐based tools, and discusses functional features that need further investigation in order to improve their performance.

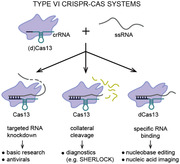

## 1 Introduction

CRISPR‐Cas systems are adaptive immune systems that protect bacteria and archaea against phages and other invasive mobile genetic elements (MGEs).^[^ ^1^, ^2^, ^3^, ^4^ ^]^ The hallmark feature of these systems is the array of genomic DNA known as clustered regularly interspaced short palindromic repeats (CRISPRs), between which memory of previous infections is stored in form of short sequences (spacers) acquired from invading MGEs.^[^ ^5^, ^6^, ^7^ ^]^ Adjoining the CRISPR array are multiple genes encoding CRISPR‐associated (Cas) proteins. Cas proteins perform different tasks in the three phases of CRISPR‐Cas‐driven immunity: adaptation, CRISPR RNA (crRNA) biogenesis, and interference.^[^ ^2^, ^3^, ^8^, ^9^, ^10^, ^11^, ^12^ ^]^ During the adaptation phase, Cas1 and Cas2 select and modify segments of foreign nucleic acids (termed protospacers), eventually adding them to the CRISPR array.^[^ ^9^, ^10^, ^12^ ^]^ Upon subsequent infection, spacers are transcribed along with the rest of CRISPR array into a long precursor crRNA (pre‐crRNA). pre‐crRNA is then processed either by Cas effectors or other endogenous endonucleases into mature crRNAs (also known as guide RNAs, gRNAs) containing a direct repeat and a spacer sequence.^[^ ^13^, ^14^ ^]^ Some systems also require a second small RNA known as transactivating crRNA, which binds with crRNA to form functional gRNA.^[^ ^14^ ^]^ gRNAs are then used by Cas interference module as a template that guides the effector to cleave complementary nucleic acid sequences in MGEs, thereby curbing infection.^[^ ^2^, ^3^, ^11^ ^]^

The unceasing arms race between prokaryotes and MGEs has led to evolutionary development of a great variety of CRISPR‐Cas systems.^[^ ^15^, ^16^, ^17^, ^18^, ^19^ ^]^ Currently known CRISPR‐Cas systems can be grouped into class 1 and class 2 systems, which are further divided into types and subtypes. Class 1 CRISPR‐Cas systems (type I, III, and IV systems) utilize a wide assortment of smaller Cas proteins to form a multi‐subunit interference complex.^[^ ^20^, ^21^ ^]^ By contrast, class 2 systems (type II, V, and VI systems) use a single, comparatively larger Cas effector protein for interference and, in certain cases, crRNA biogenesis.^[^ ^22^, ^23^, ^24^ ^]^

In the years following the discovery of Cas9, the DNA‐targeting type II and type V CRISPR‐Cas effectors have been successfully harnessed for a variety of applications in genome editing and nucleic acid detection.^[^ ^25^, ^26^, ^27^, ^28^, ^29^, ^30^, ^31^, ^32^, ^33^ ^]^ Engineered and certain naturally occurring variants of Cas9 have also been used for RNA targeting and binding, thus initiating the use of CRISPR‐Cas technology in RNA manipulation and potentially advancing this field by overcoming limitations of conventionally used RNA‐targeting methods.^[^ ^34^, ^35^ ^]^ However, the fact that Cas9 also retains its ability to target DNA involves risk from undesired off‐target effects on genes.^[^ ^36^ ^]^ Moreover, Cas9 effectors may not necessarily be efficient in all RNA‐targeting applications, so expanding the CRISPR‐Cas toolbox with new and diverse effectors would allow greater flexibility and more sophisticated RNA manipulation.

**Figure 1:**
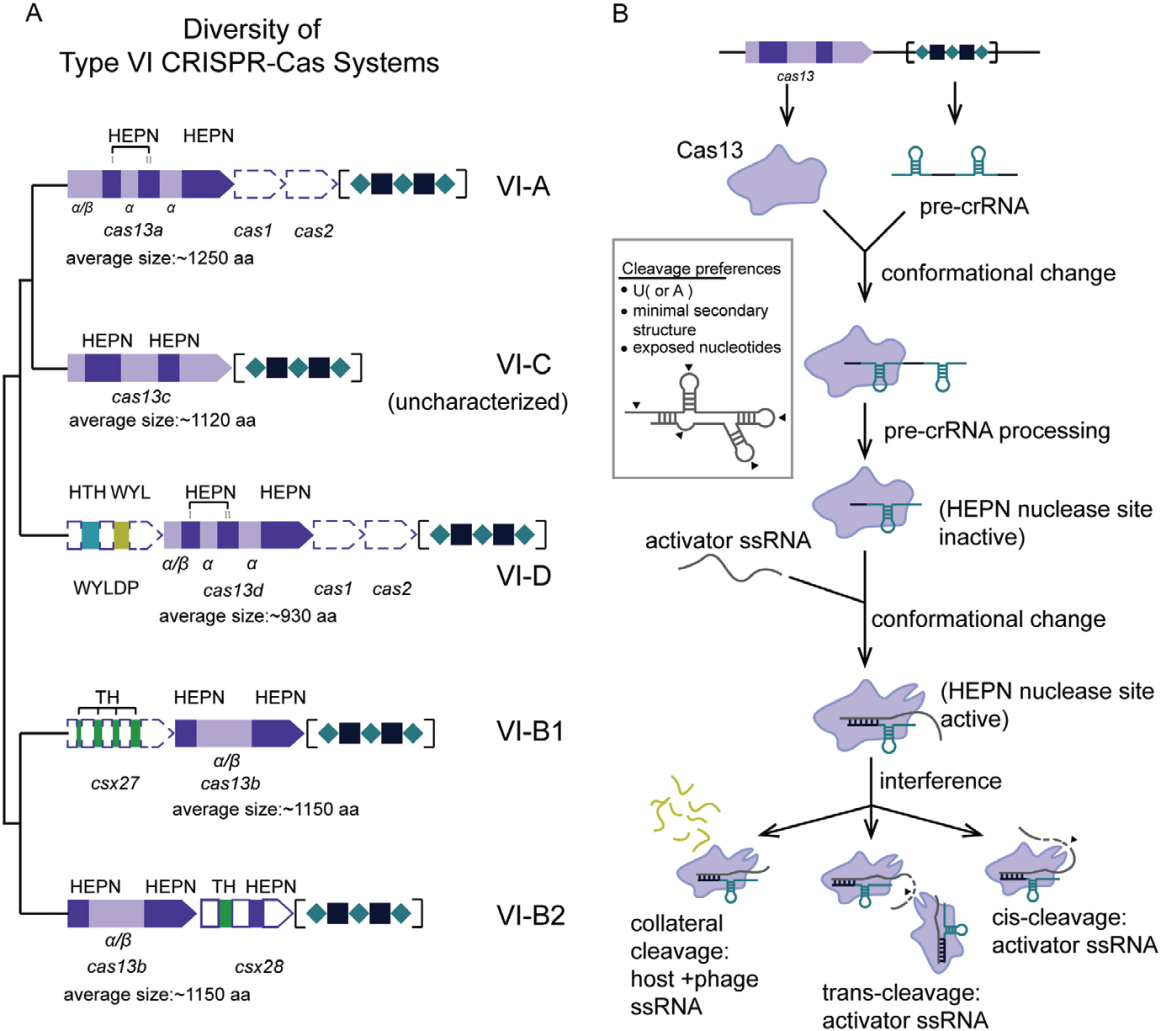
Diversity and functional characteristics of type VI CRISPR‐Cas systems. A) Architecture of CRISPR loci of known subtype of type VI CRISPR‐Cas systems and their phylogenetic relationships. The phylogenetic tree is adopted from refs. ^[^ ^53^, ^56^ ^]^ for illustrative purposes and does not display accurate branch lengths and evolutionary distances. The approximate locations of the two HEPN domains within each Cas effector gene are indicated with dark purple, and the secondary structure composition of the remainder of the gene sequence (light purple) is denoted below the corresponding fragment of the gene (*α* indicates an *α*‐helical structure, whereas *α*/*β* indicates a mixed *α*‐helical and *β*‐sheet structure). The average sizes of each Cas effector are indicated underneath the gene cartoon. The protein‐encoding gene sequences indicated with dashed lines (i.e., Cas1, Cas2 genes in type VI‐A and VI‐D systems, WYL‐domain containing accessory protein in type VI‐D systems and Csx27 accessory protein in type VI‐B1 systems) are not present in all CRISPR loci. CRISPR sequences are denoted with alternating teal rhombuses (direct repeat sequences) and black squares (spacer sequences) in square brackets. The architecture of the type VI‐C CRISPR loci is incomplete as it remains uncharacterized. Gene cartoons are not shown in scale. Abbreviations: HEPN, higher eukaryotes and prokaryotes‐binding domain; WYLDP, WYL domain‐containing protein; HTH, helix‐turn‐helix domain; WYL, WYL domain; TH, putative transmembrane helices. B) General schematic representing functions of type VI CRISPR‐Cas effectors. Upon phage infection, the host bacteria express Cas13 effectors and transcribe the CRISPR sequence into a long precursor CRISPR RNA (pre‐crRNA). Cas13 effectors bind pre‐crRNA and process it autonomously into mature crRNAs, thus forming a Cas13‐crRNA immune surveillance complex. Once the Cas13‐crRNA complex binds a complementary single‐stranded RNA of phage origin (activator ssRNA), hybridization between crRNA spacer and ssRNA is initiated, which is accompanied by a gradual conformational change within the Cas13 effector that eventually results in activation of the HEPN nuclease site (located on the outer surface of the protein). Using Mg^2+^ as a cofactor, the Cas13 effector (i.e., HEPN nuclease site) activated in this manner subsequently engages in RNA interference, cleaving nonspecifically bound activator ssRNA in cis and other available ssRNAs (both phage and host ssRNA) in trans. Cleavage preferences of Cas13 effectors are shown in the box on the left side of the panel.

More recently, type VI CRISPR‐Cas systems that exclusively target single‐stranded RNA (ssRNA) have been discovered.^[^ ^37^, ^38^, ^39^, ^40^ ^]^ Four subtypes (A–D) have been identified to date, of which subtypes VI‐A, VI‐B, and VI‐D have been functionally characterized along with respective effectors, i.e., Cas13a, Cas13b, and Cas13d (**Figure** **1**A and **Table** **1**). Functionally, all Cas13 effectors are crRNA‐guided RNases with two distinct and independent catalytic centers. One catalytic center processes pre‐crRNA, and the other is formed by two R‐X_4_‐H motifs typical of higher eukaryotes and prokaryotes nucleotide‐binding (HEPN) domains that mediate ssRNA cleavage. As opposed to Cas9 that specifically cleaves crRNA spacer‐complementary dsDNA sequences (i.e., target DNA) in cis, the activated Cas13‐crRNA interference complex cleaves nonspecifically both the crRNA‐bound complementary ssRNA sequence (henceforth referred to as activator RNA) in cis and any other encountered RNA (both host and viral RNA) in trans (also known as collateral or bystander cleavage) (Figure 1B).^[^ ^37^, ^38^, ^39^, ^40^ ^]^ The cleavage preferentially occurs within structurally exposed regions of the RNA secondary structures, usually at uridine (U) or adenosine (A) (Figure 1B). Given their potent performance in mammalian cells, Cas13 effectors have already been used as tools for RNA manipulation, albeit their practical application is still in its infancy.^[^ ^38^, ^39^, ^41^, ^42^, ^43^, ^44^, ^45^, ^46^, ^47^, ^48^, ^49^ ^]^

**Table 1:** Comparison of type VI CRISPR‐Cas systems

	VI‐A	VI‐B	VI‐D
**Cas effector**	Cas13a	Cas13b	Cas13d
size	≈1250 aa	≈1150 aa	≈930 aa
architecture	REC and NUC lobe	pyramidal (binary complex)	REC and NUC lobe
pre‐crRNA processing site	Helical‐1 and HEPN‐2 domains	RRI‐2 domain (Lid domain)	HEPN‐2 domain
pre‐crRNA processing mechanism	acid–base	acid–base	acid–base
RNase catalytic site	surface‐exposed	surface‐exposed	surface‐exposed
ssRNA cleavage preferences	U‐ and A‐cleaving effectors	pyrimidine bases (mostly U)	U
protospacer‐flanking sequence^a)^	5′ non‐G^b)^	5′ non‐C 3′ NAN or NNA	no restrictions
**small accessory proteins**	none	Csx27 and Csx28^c)^	WYL domain‐containing protein^d)^
**crRNA**			
orientation (repeat→spacer)	5′→3′	3′→5′	5′→3′
repeat length (mature)	varies, 27–32 nt	36 nt (short) 88 nt (long)^e)^	30 nt
repeat architecture	stem‐loop	distorted stem‐loop (four subregions)	stem‐loop
recognition by Cas13 effector	sequence‐ and structure‐specific	structure‐specific	sequence‐ and structure‐ specific
spacer mismatch‐sensitive regions	central seed region HEPN nuclease switch region	central region	3′ end region internal region^f)^

^a)^ cleavage appears to be unrestricted or negligibly affected by protospacer‐flanking sequences for certain orthologs or under certain conditions

^b)^ confirmed in U‐cleaving Cas13a orthologs

^c)^ Csx27 represses Cas13b activity and is found inconsistently in type VI‐B1 CRISPR loci, whereas, whereas Csx28 enhances Cas13b activity and is universally present in type VI‐B2 loci; both accessory proteins can regulate orthogonal Cas13b effectors

^d)^ WYL domain‐containing protein enhances Cas13d activity and is found inconsistently in type VI‐D CRISPR loci

^e)^ consists of 5′ and 3′ fragments of the 36‐nt repeat sequence separated by an intervening repeat region

^f)^ may vary between orthologs.

In the light of recent studies on Cas13 effectors, this review article will summarize the current knowledge of structural and mechanistic basis for function of Cas13 effectors, outline the reported applications and point out issues that need to be addressed before these effectors could be used more broadly and efficiently.

## 2 Cas13a

Cas13a (formerly known as Class 2 candidate 2, C2c2) and the associated type VI‐A CRISPR‐Cas systems were first discovered by Shmakov et al. via computational pipeline that searched for candidate CRISPR‐Cas loci among microbial genomic and metagenomic data.^[^ ^37^ ^]^ Contrary to the previous knowledge of CRISPR‐Cas loci, a number of identified type VI‐A loci lack *cas1* and *cas2* genes, containing only a CRISPR array and a Cas13 effector gene sequence (Figure 1A).^[^ ^37^ ^]^ The CRISPR arrays of type VI‐A systems are markedly unstructured and heterogeneous, with direct repeat lengths ranging from 35 to 39 bp.^[^ ^37^ ^]^ In spite of the seeming heterogeneity, type VI‐A CRISPR repeats can be assigned to two groups with respect to functional exchangeability of noncognate crRNAs between orthogonal Cas13a systems.^[^ ^50^ ^]^

Cas13a is a large protein (>1000 aa) and currently the most well‐understood type VI effector. Its primary sequence lacks appreciable similarity to other known Cas effectors.^[^ ^37^ ^]^ Currently known Cas13a orthologs are grouped into two subfamilies (U‐cleaving and A‐cleaving) based on their cleavage preferences and the aforementioned functional exchangeability of noncognate crRNAs.^[^ ^50^ ^]^ Activation of ssRNA cleavage appears to be restricted, at least for certain orthologs, by a 3′‐H (non‐G) protospacer flanking sequence (PFS).^[^ ^50^, ^51^ ^]^ Pre‐crRNA processing is not a prerequisite for RNA interference, but enhances the activity by releasing crRNAs from CRISPR array, thus allowing optimal use of individual crRNA guide sequences.^[^ ^50^ ^]^

### 2.1 Domain Organization

To date, several structures of Cas13a homologs and their complexes with crRNA and/or activator RNA have been reported: (1) apo protein and binary complex of the U‐cleaving *Leptotrichia shahii* Cas13a (LshCas13a),^[^ ^52^ ^]^ (2) binary and ternary complex of the U‐cleaving *Leptotrichia buccalis* Cas13a (LbuCas13a),^[^ ^53^ ^]^ and (3) binary complexes containing pre‐crRNA/crRNA of the A‐cleaving *Lahnospiraceae bacterium* Cas13a (LbaCas13a).^[^ ^54^ ^]^

**Figure 2:**
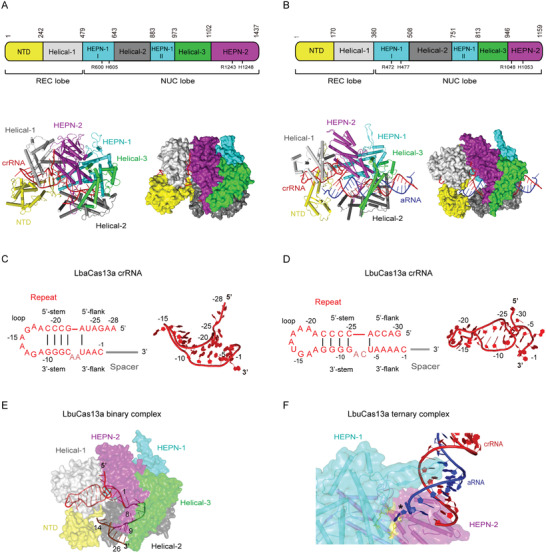
Structural features of type VI‐A CRISPR‐Cas effectors. All structural figures were generated in PyMOL (http://pymol.org). A,B) Top: linear domain organization of *Lahnospiraceae bacterium*Cas13a (LbaCas13a) (A) and *Leptotrichia buccalis* Cas13a (LbuCas13a) (B) with the HEPN nuclease active site residues, REC and NUC lobes annotated. All figures use the same color code unless otherwise stated. Bottom: cartoon and surface representations of the overall structure of the LbaCas13a‐crRNA binary complex (PDB ID: 5W1H)(A) and LbuCas13a‐crRNA‐aRNA ternary complex (PDB ID: 5XWP)(B). Domains are colored according to the linear domain organization diagram, crRNA is colored red and activator RNA (aRNA) is colored blue. C,D) Left: diagram of the LbaCas13a‐crRNA (C) and LbuCas13a‐crRNA (D) secondary structure. Because the focus of the diagram is structure and sequence of the repeat region, spacer nucleotides were omitted and spacer region is represented by a gray line. Subregions of the repeat region are annotated, and Watson–Crick base pairs are denoted with black lines. Dinucleotide bulge residues are indicated in a lighter shade of red. Right: 3D structure of the crRNA repeat region from the LbaCas13a‐crRNA binary complex (PDB ID: 5W1H)(C) and LbuCas13a‐crRNA‐aRNA ternary complex (PDB ID: 5XWP)(D). Nucleotides belonging to repeat region are colored and annotated according to the diagram on the left, whereas spacer nucleotides were entirely omitted. E) View of conformations and locations of crRNA spacer subregions in LbuCas13a‐crRNA binary complex (PDB ID: 5XWY). LbuCas13a is shown as partially transparent surface representation in order to distinguish concealed and solvent‐exposed subregions of crRNA. Domains of LbuCas13a are colored according to panel (B), crRNA repeat region is colored red, and spacer is colored brown. Marked nucleotides denote borders of each spacer subregion: nucleotides 1–8 correspond to the conformationally distorted 5′ part of spacer located in NUC lobe, whereas nucleotides 9–14 and 15–26 correspond to the solvent‐exposed central seed region and the 3′ part of spacer, respectively. F) View of the interactions between aRNA and LbuCas13a HEPN nuclease active site. The active‐site proximal *β*‐hairpin from HEPN‐1 domain extends into a helical groove formed by the crRNA‐aRNA duplex, guiding the 5′‐terminal nucleotide of aRNA into the pocket in which the active site is located. crRNA is colored red, aRNA is colored blue, and LbuCas13a is shown as a mixed surface‐cartoon representation with partially transparent surface. The active site residues R472, H477, R1048A, and H1053A are colored yellow and the 5′‐terminal nucleotide of aRNA is indicated with asterisk (*).

Similarly to type II and V CRISR‐Cas effectors, Cas13a is predominantly *α*‐helical and comprises two lobes, namely, the crRNA recognition (REC) lobe and the nuclease (NUC) lobe.^[^ ^52^, ^53^, ^54^ ^]^ However, its structure highly differs at architectural and domain organization level (**Figure** **2**A,B). The REC lobe is divided into N‐terminal domain (NTD) and Helical‐1 domain, between which a highly basic cleft accommodating crRNA repeat region is formed (Figure 2A,B). The NUC lobe consists of HEPN‐1 domain, Helical‐2 domain, HEPN‐2 domain, and Helical‐3 domain (also termed Linker domain) that interconnects HEPN‐1 and HEPN‐2 domains (Figure 2A,B). HEPN‐1 domain is divided into two subdomains (HEPN‐1 I and HEPN‐1 II) by the Helical‐2 domain.^[^ ^52^, ^53^, ^54^ ^]^ The two R‐X_4_‐H motifs forming the HEPN nuclease site are located in the outer surface groove formed by HEPN‐1 I and HEPN‐2 domains.

### 2.2 Structure and Recognition of crRNA Repeat Region

Repeat region of mature type VI‐A crRNAs (often referred to as 5′ handle) adopts a comparatively simple structure divided into a stem‐loop with a 3′ dinucleotide bulge, a 5′ and a 3′ single‐stranded flanking region (Figure 2C,D).^[^ ^52^, ^53^, ^54^, ^55^ ^]^ All of these subregions have distinct subfamily‐specific characteristics in terms of sequence, length, and conformation, and therefore cannot be used interchangeably between the two Cas13a subfamilies.^[^ ^50^, ^52^, ^54^ ^]^ For instance, one crucial distinction between crRNA repeat regions is the sequence of the dinucleotide bulge, which reads AC in the U‐cleaving systems and AA in the A‐cleaving systems.^[^ ^52^, ^54^ ^]^ crRNA repeat region is generally intolerant to most sequence modifications, including base changes, altering the length of the stem region, inversion of the stem‐loop sequence and shortening of the loop.^[^ ^52^, ^55^ ^]^ Single mutations of the dinucleotide bulge or interacting residues abolish both RNA cleavage and pre‐crRNA processing, which underscores the importance of the dinucleotide bulge in crRNA recognition and thereby both RNase activities of Cas13a.^[^ ^52^ ^]^

Upon crRNA binding, Cas13a undergoes conformational rearrangement into a more compact structure that stabilizes the binary complex, closes the crRNA‐binding channel, and facilitates activator RNA binding.^[^ ^52^ ^]^ crRNA repeat region is recognized by Cas13a in sequence‐specific and structure‐specific manner in both A‐cleaving and U‐cleaving Cas13a effectors.^[^ ^52^, ^54^ ^]^

### 2.3 Pre‐crRNA Processing

Critical catalytic residues for pre‐crRNA processing have been determined for all three Cas13a orthologs with reported structures, but more detailed insight into the underlying acid‐base mechanism have been given for the A‐cleaving LbaCas13a.^[^ ^52^, ^53^, ^54^ ^]^ Although all Cas13a effectors are likely to use an acid–‐base mechanism for pre‐crRNA processing, the exact way in which pre‐crRNA is cleaved may somewhat differ between the A‐cleaving and U‐cleaving orthologs because of the distinct sets of residues involved in pre‐crRNA processing.^[^ ^52^, ^53^, ^54^ ^]^ Besides differences in primary structures of Cas13a orthologs, divergence in mechanisms for pre‐crRNA processing is also preconditioned by the subfamily‐specific 5′ flanking sequence of their crRNAs, particularly the two cleavage site‐proximal nucleotides which are recognized in nucleobase‐specific manner^.[^ ^50^, ^52^, ^53^, ^54^ ^]^ Moreover, although it belongs to the U‐cleaving subfamily, LshCas13a employs a distinct pre‐crRNA cleavage site to generate mature crRNA with 5′ flanking region shorter than other U‐cleaving Cas13a orthologs.^[^ ^50^, ^52^, ^53^ ^]^

### 2.4 Recognition of crRNA Spacer Region

As effector of adaptive RNA‐guided immunity, Cas13a is capable of effectively utilizing crRNAs with spacers of varied sequence identity and lengths ranging from 20 to 28 nucleotides.^[^ ^51^, ^52^, ^53^, ^54^, ^55^ ^]^ In the available binary complex structures, the 5′ part of spacer is concealed in the NUC lobe cavity and adopts distorted conformation stabilized by extensive sugar–phosphate backbone interactions (Figure 2E).^[^ ^52^, ^53^, ^54^ ^]^ The central part of the spacer emerges from the NUC lobe cavity and traverses a solvent‐exposed groove formed between Helical‐2 and NTD domains (Figure 2E).^[^ ^52^, ^53^, ^54^ ^]^ Although they are not visible in all orthologs, current data indicate that the central and 3′ part of spacer maintain a near A‐form helical configuration in Cas13a‐crRNA binary complexes with reported structures.^[^ ^53^, ^54^ ^]^ Therefore, the low visibility (i.e., low electron density) is likely due to high flexibility of the solvent‐exposed, ordered spacer segment lacking stable interactions with Cas13a. The central and 3′ parts of spacer are thus tethered to protein and can probe nearby solvent environment for potential activator RNA molecules (Figure 2E). Indeed, the central part of spacer was confirmed as the seed region by biochemical studies.^[^ ^51^, ^52^, ^53^, ^54^, ^56^ ^]^ The central seed region of type VI‐A systems is in stark contrast with other RNA‐guided nucleases (e.g., type II and type V CRISPR‐Cas effectors) that utilize solvent‐accessible preordered protein‐bound seed regions, which is energetically favorable for target search, mismatch discrimination, and target‐guide duplex formation.^[^ ^54^, ^57^, ^58^, ^59^, ^60^, ^61^ ^]^ Considering that the entropic cost for target search and duplex formation is comparatively higher in case of a dynamic seed region and inaccessible distorted 5′ part of spacer, it is unclear how Cas13 effectors overcome additional energetic barriers during ternary complex formation.^[^ ^54^ ^]^ Nevertheless, it is likely that cytotoxic effects of nonspecific RNA interference necessitated evolutionary development of a more rigorous mechanism for HEPN nuclease site activation.

### 2.5 Activator RNA Binding, HEPN Nuclease Site Activation and RNA Interference

Once a potential activator RNA binds the central seed region of crRNA, the process of sequence interrogation and crRNA spacer‐activator RNA duplex propagation is initiated.^[^ ^52^, ^53^, ^54^, ^56^ ^]^ Although absolute complementarity is not necessary as a means to maintain immunity against rapidly mutating bacteriophages, two regions of spacer are particularly sensitive to mismatches: the central seed region (spacer nucleotides 9–14 in LbuCas13a) crucial for binding to activator RNA and the “HEPN nuclease switch” region (spacer nucleotides 5–8 in LbuCas13a) essential for triggering formation of catalytically competent HEPN nuclease active site.^[^ ^56^ ^]^ Base pairing between activator RNA and the remainder of spacer is required to further stabilize the duplex.^[^ ^56^ ^]^ The advance of sequence interrogation and crRNA spacer‐activator RNA duplex propagation gradually drives synergistic conformational changes in both crRNA and Cas13a, which ultimately results in a ternary complex with fully activated HEPN nuclease site ready to engage in RNA interference. These conformational changes expand the binding channel to accommodate the crRNA‐activator RNA duplex, enable interactions between the duplex and NUC lobe, and activate the HEPN nuclease active site by exposing the obscured R‐X_4_‐H motif of HEPN‐1 domain to the surface and bringing it closer to the R‐X_4_‐H motif in HEPN‐2 domain.^[^ ^52^, ^53^, ^54^, ^56^ ^]^ Most relevant insights into these conformational changes are provided by the structure of LbuCas13a ternary complex and its comparison to the structure of LbuCas13a binary complex.^[^ ^53^ ^]^

In contrast to the composite conformation in binary complex, the activator RNA‐bound 28‐nt crRNA spacer in the LbuCas13a ternary complex approximates to a regular A‐form helix throughout its length (Figure 2B).^[^ ^53^ ^]^ Base pairs 1–24 of the duplex are bound within the positively charged central channel in NUC lobe and make contacts primarily with Helical‐2, Helical‐3, and HEPN‐1 domains.^[^ ^53^ ^]^ LbuCas13a mainly interacts with the sugar‐phosphate backbone of nucleotides 7–15 (generally corresponds to seed region and parts of the HEPN switch region) and 18–24 of crRNA spacer and nucleotides 11–21 of activator RNA.^[^ ^53^ ^]^ Numerous spacer‐interacting LbuCas13a residues are important for the ssRNA cleavage activity.^[^ ^53^ ^]^ The duplex beyond 24th base pair is located outside of the protein, explaining why Cas13a effectors can use spacers longer than 20 nucleotides with similar efficiency.^[^ ^53^ ^]^

RNA interference by Cas13a effectors is divalent metal ion‐dependent and cleaves ssRNA sequences nonspecifically both in cis and in trans.^[^ ^51^, ^52^, ^53^, ^54^, ^55^ ^]^ Unlike catalytic centers of type II and V effectors that are buried within protein in proximity to target DNA‐guide RNA duplex, the HEPN nuclease active site of type VI effectors is situated in a groove at the outer surface of the protein, distant from the positively charged central channel that binds the crRNA‐activator RNA duplex.^[^ ^52^, ^53^, ^54^ ^]^ The activator RNA bound by the effector therefore needs to be sufficiently long for cleavage in cis, but the catalytic center can be easily accessed by any RNA in solution for cleavage in trans. In the LbuCas13a ternary complex, the 5′ terminal nucleotide of activator RNA swings away from the crRNA‐activator RNA duplex and inserts into the HEPN nuclease active center of the adjacent LbuCas13a within the same asymmetric unit, revealing how HEPN nuclease site in Cas13a interacts with RNA molecules during RNA interference (Figure 2F).^[^ ^53^ ^]^ RNA is captured in the close vicinity of the catalytic site by an HEPN‐1 *β*‐hairpin, which extends into major groove of the crRNA‐activator RNA duplex and contacts it through van der Waals interactions (Figure 2F).^[^ ^53^ ^]^ Substitution of active site residues that interact with the 5′ terminal nucleotide of activator RNA as well as truncation or deletion of the *β*‐hairpin reduces both in cis and in trans RNA interference activity, which indicates its significance in capturing ssRNA.^[^ ^53^ ^]^

## 3 Cas13b

Insights from previous computational pipelines such as the fact that the presence of *cas1* and *cas2* genes in CRISPR‐Cas loci is not essential allowed design of a new computational discovery pipeline used in 2016 to identify Cas13b, the ≈1100–1200‐amino acid effector of type VI‐B systems.^[^ ^37^, ^38^ ^]^ Although their loci lack an adaptation module, most type VI‐B systems originate from bacterial hosts that possess another CRISPR‐Cas locus with *cas1* and *cas2* genes, suggesting that type VI‐B systems acquire spacers in trans.^[^ ^38^ ^]^

Type VI‐B systems possess a number of distinct features.^[^ ^38^ ^]^ First, crRNA direct repeat sequences are conserved with regard to their size (36 nt), sequence and predicted structure.^[^ ^38^ ^]^ Second, the processed mature crRNA adopts orientation opposite from Cas13a and Cas13d crRNAs.^[^ ^38^ ^]^ Furthermore, mature crRNAs are generated in two variants from distinct parts of CRISPR locus, i.e., the short crRNA (66 nt) containing a single 36 nt direct repeat and the long crRNA (118 nt) containing a composite repeat region formed by 5′ and 3′ fragments of the 36 nt direct repeat sequence separated by an intervening repeat sequence.^[^ ^38^ ^]^ Third, Cas13b‐mediated interference is restricted by two PFSs, namely, the 5′ non‐C and the 3′ NAN or NNA.^[^ ^38^ ^]^ Fourth, ssRNA cleavage occurs at pyrimidine base, with preference for uracils.^[^ ^38^ ^]^ Fifth, Cas13b interference is regulated by one of two small accessory proteins (≈200 aa) with one or more transmembrane helices (Figure 1A): subtype VI‐B1 systems inconsistently contain Csx27 that represses Cas13b‐mediated cleavage, whereas subtype VI‐B2 systems universally contain Csx28 that enhances Cas13b‐mediated cleavage. Both accessory proteins seem to be capable of regulating orthogonal type VI‐B systems, which expands their utility.^[^ ^38^ ^]^ A comparative genomic analysis study published in 2019 suggested that Csx27 is a member of transmembrane protein family that may function as a component of membrane‐linked ssDNA uptake machinery and/or DNA modification system functionally associated with ubiquitin system components and WYL domain‐containing proteins (which also play roles of accessory proteins in type I and type VI‐D CRISPR‐Cas systems) for host defense against foreign ssDNA.^[^ ^62^ ^]^ However, how Csx27 would interconnect these machineries for defense against foreign ssDNA with Cas13b and how Csx27 and Csx28 exactly modulate the activity of Cas13b needs further investigation.

### 3.1 Domain Organization

**Figure 3:**
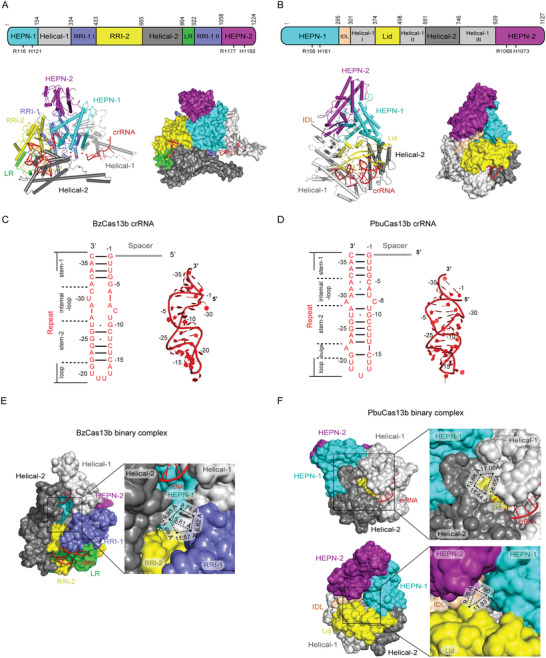
Structural features of type VI‐B CRISPR‐Cas effectors. All PyMOL figures were generated using the available structures of *Bergeyella zoohelcum* Cas13b (BzCas13b)‐crRNA binary complex (PDB ID: 6AAY) and *Prevotella buccae* Cas13b (PbuCas13b)‐crRNA binary complex (PDB ID: 6DTD). A,B) Top: linear domain organization of BzCas13b (A) and PbuCas13b (B) with the HEPN nuclease active site residues annotated. All figures use the same color code. Bottom: cartoon and surface representations of the overall structure of the BzCas13b‐crRNA binary complex (A) and PbuCas13b‐crRNA binary complex (B). Domains are colored according to the linear domain organization diagram and crRNA is colored red. C,D) Left: diagram of the BzCas13b‐crRNA (C) and PbuCas13b‐crRNA (D) secondary structure. Because the focus of the diagram is structure and sequence of the repeat region, spacer nucleotides were omitted and spacer region is represented by a gray line. Subregions of the repeat region are annotated, Watson–Crick base pairs are denoted with black lines, whereas non‐Watson–Crick base pairing is denoted with a black dot. Right: 3D structure of the crRNA repeat region from the BzCas13b‐crRNA binary complex (C) and PbuCas13b‐crRNA binary complex (D). Nucleotides belonging to repeat region are colored and annotated according to the diagram on the left, whereas spacer nucleotides were entirely omitted. E) Close‐up view of the opening on BzCas13b leading into the positively charged central cavity. The surface representation was set to solvent‐accessible mode for clarity and dimensions of the opening are given for comparison with the two openings on PbuCas13b. F) Top: close‐up view of the larger opening on PbuCas13b leading into the positively charged central cavity. Bottom: close‐up view of the smaller opening on BzCas13b leading into the positively charged central cavity. The surface representation was set to solvent‐accessible mode for clarity and dimensions of the openings are given for comparison with the opening on BzCas13b.

Recently, the structures of subtype VI‐B1 *Berg* *e* *yella zoohelcum* (Bz) and subtype VI‐B2 *Prevotella buccae* (Pbu) Cas13b‐crRNA binary complexes reported by our and Zhang's research group provided deeper insight into mechanisms underlying Cas13b activity (**Figure** **3**A,B).^[^ ^63^, ^64^ ^]^ In both structures, Cas13b assumes a unique pyramidal shape with positively charged central cavity that accommodates crRNA (Figure 3A,B).^[^ ^63^, ^64^ ^]^ The bilobed architecture typical of class 2 CRISPR‐Cas effectors is not discernible in the structure of Cas13b‐crRNA binary complex, and its domain organization substantially differs from other type VI effectors. HEPN‐1 and HEPN‐2 domains respectively comprise the N‐ and C‐extremities of the protein primary structure, but Cas13b folds in such way that HEPN‐2 rests on top of HEPN‐1 so that the two R‐X_4_‐H motifs are in relative proximity to each other (Figure 3A,B).^[^ ^63^, ^64^ ^]^

The remainder of Cas13b is divided into a number of other domains, three in PbuCas13b and five in BzCas13b, because the authors of the two articles took different approaches in defining domain organization (Figure 3A,B).^[^ ^63^, ^64^ ^]^ Both BzCas13b and PbuCas13b have a large positively charged channel that is located at the bottom of the pyramidal structure and connects the internal cavity and solvent.^[^ ^63^, ^64^ ^]^ In BzCas13b, this channel is flanked by two long *α*‐helices from Helical‐1 and Helical‐2 domains that protrude from the protein bulk in a manner akin to pincer (Figure 3A).^[^ ^63^ ^]^ Since these two long *α*‐helices are absent in PbuCas13b, it would be interesting to determine their function and whether they are specifically associated with subtype VI‐B1 systems.

### 3.2 crRNA Recognition and Processing

The mature crRNA adopts an L‐shaped structure, with the bulk of the repeat region and 3′‐segment of spacer region shielded within the protein.^[^ ^63^ ^]^ The 36 nt repeat region forms a distorted hairpin loop roughly perpendicular to the direction of the spacer (Figure 3C,D).^[^ ^63^, ^64^ ^]^ Four subregions are identifiable in the repeat region: stem‐1, internal loop, stem‐2, and a U‐rich loop.^[^ ^63^, ^64^ ^]^ Intramolecular interactions are maintained mainly through Watson–Crick base pairing, with the addition of several hydrogen bonds and noncanonical wobble pairing (Figure 3C,D).^[^ ^63^, ^64^ ^]^ Furthermore, several nucleotides are flipped out in the repeat regions of both crRNAs, albeit these nucleotides largely differ between the two crRNAs, with the exception of C(‐8) (Figure 3C,D). crRNA makes extensive intermolecular interactions with all domains of Cas13b except HEPN‐2, most of them being with the RRI‐2 domain (Lid and Helical‐1 III in PbuCas13b).^[^ ^63^, ^64^ ^]^ Interactions between Cas13b and crRNA repeat region are generally ortholog‐specific, but the majority of interactions play role in stabilizing the phosphate backbone of crRNA, suggesting that crRNA repeat region of type VI‐B systems is recognized in structure‐specific rather than sequence‐specific manner.^[^ ^63^, ^64^ ^]^

In BzCas13b, the 22 nt spacer region is located within the positively charged cavity and wrapped by Helical‐1, HEPN‐1, RRI‐1, and RRI‐2 domains on one side and Helical‐2 domain on the other side.^[^ ^63^ ^]^ The central part of spacer (nucleotides 9–15) is not visible due to its high flexibility.^[^ ^63^ ^]^ Spacer nucleotides 16–22 are anchored within a surface‐exposed groove formed between the long *α*‐helix and the bulk of Helical‐1 domain. Although achieved by distinct sets of interactions, both BzCas13b and PbuCas13b adjust the spacer direction relative to repeat region by obstructing the movement of the first spacer nucleotide.^[^ ^63^ ^]^ This suggests that correct positioning of the first spacer nucleotide is important for determining spacer trajectory and efficient activator RNA binding by both Cas13b orthologs.

Pre‐crRNA processing is executed through base‐catalyzed hydrolysis by the Lid domain, which uses strictly conserved arginine and lysine residues to process pre‐crRNA downstream of the 3′‐terminal nucleotide A(‐37).^[^ ^63^, ^64^ ^]^ Given that the nucleotide A(‐37) originates from the 3′‐adjacent spacer region and not from CRISPR direct repeat, it is not recognized in base‐specific manner by the catalytic residues.^[^ ^63^ ^]^ Our unpublished cryo‐EM structure of PbuCas13b‐crRNA binary complex indicates that PbuCas13b processes pre‐crRNA in the same way as BzCas13b, i.e., by cleaving downstream of the repeat region‐proximal nucleotide belonging to the 3′‐adjacent spacer region.

### 3.3 Mechanism for Activator RNA Binding

On the basis of their structural and functional data, Slaymaker et al. proposed a model for Cas13b‐mediated activator RNA binding in which the crRNA‐bound Cas13b first uses the repeat region‐proximal (3′‐end) of the spacer to probe ssRNA.^[^ ^64^ ^]^ In case of complementarity, the initial binding of potential activator RNA to the crRNA spacer induces opening of HEPN‐1 and Helical‐2 domains to allow the RNA access into to the positively charged central cavity. The remainder of the RNA sequence is then scanned for full complementarity with spacer before full conformational activation of the bipartite HEPN site is achieved.^[^ ^64^ ^]^ However, the structure of BzCas13b‐crRNA binary complex lacks the channel between the HEPN‐1 and Helical‐2 domains proposed as the route for activator RNA into the central cavity.^[^ ^63^, ^64^ ^]^ In addition, this channel in PbuCas13b is smaller than the positively charged channel and may not be able to widen enough to accept activator RNA since HEPN‐1 and Helical‐1 domains are interconnected with the interdomain linker that likely hinders larger movements of HEPN‐1 domain (Figure 3E,F). Furthermore, the 3′‐end of spacer is buried within protein in both BzCas13b and PbuCas13b, whereas in the BzCas13b‐crRNA binary complex the central part of the spacer (nucleotides 9–15) traverses the large solvent‐accessible channel similarly to central seed regions of type VI‐A systems (Figure 3A).^[^ ^63^ ^]^ In line with that, while tandem mismatches are not tolerated along the length of spacer, a single mismatch in the central part of spacer (nucleotides 12–17) is sufficient to abolish HEPN nuclease activity of PbuCas13a.^[^ ^64^ ^]^ Therefore, activator RNA is likely first probed by the central part of the spacer, after which activator RNA binding proceeds toward the 5′ and 3′ ends of the spacer.

## 4 Cas13d

**Figure 4:**
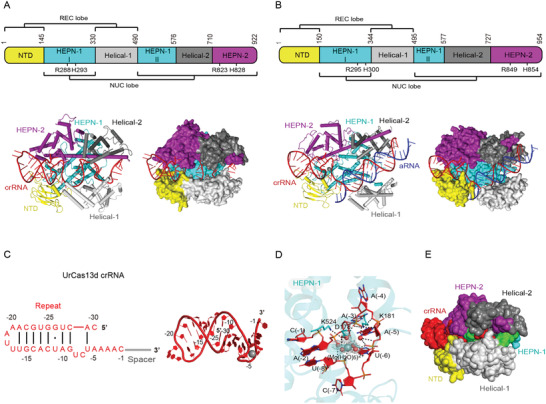
Structural features of type VI‐D CRISPR‐Cas effectors. All PyMOL figures were generated using the available structures of uncultured *Ruminococcus* sp. Cas13d (UrCas13d)‐crRNA binary complex (PDB ID: 6IV9) and *Eubacterium siraeum* Cas13d (EsCas13d)‐crRNA‐aRNA ternary complex (PDB ID: 6E9F). A,B) Top: linear domain organization of UrCas13d (A) and EsCas13d (B) with the HEPN nuclease active site residues, REC and NUC lobes annotated. All UrCas13d figures use the same color code. Bottom: cartoon and surface representations of the overall structure of the UrCas13d‐crRNA binary complex (A) and EsCas13d‐crRNA‐aRNA ternary complex (B). Domains are colored according to the linear domain organization diagram, crRNA is colored red and activator RNA (aRNA) is colored blue. C) Left: diagram of the UrCas13d crRNA secondary structure. Because the focus of the diagram is structure and sequence of the repeat region, spacer nucleotides were omitted and spacer region is represented by a gray line. Watson–Crick base pairs are denoted with black lines, whereas non‐Watson–Crick base pairing is denoted with a black dot. Right: 3D structure of the crRNA from the UrCas13d‐crRNA binary complex. Nucleotides belonging to repeat region are colored and annotated according to the diagram on the left, whereas spacer nucleotides were entirely omitted. The pentahydrated magnesium ion interacting with crRNA repeat region is shown as a gray sphere, but the water molecules were omitted. D) Detailed view of interactions between the pentahydrated magnesium ion and crRNA repeat region in the UrCas13d‐crRNA binary complex. UrCas13d residues participating in the interaction are shown as sticks, and hydrogen bonds are indicated with black dashed lines. The 2Fo–Fc omit maps for the pentahydrated magnesium are contoured at 0.4 *σ* level. The figure was generated anew based on Figure 2b in Zhang et al.^[^ ^66^ ^]^ E) Surface representation of the UrCas13d‐crRNA binary complex displaying solvent accessibility of crRNA spacer region. Two mismatch‐sensitive regions of crRNA spacer (nucleotide positions 5–8 and 13–22) are colored green and the remainder of crRNA is colored red. UrCas13d is colored consistently with the linear domain organization diagram in panel (A). The surface representation was set to solvent‐accessible mode for clarity.

With the approximate size of 930 amino acids, Cas13d is currently the smallest identified type VI CRISPR‐Cas effector.^[^ ^39^, ^40^ ^]^ Cas13d was discovered by further improving the search strategies of previous computational pipelines and expanding the search to smaller effectors.^[^ ^39^, ^40^ ^]^ Outside the two HEPN domains, the primary structure of Cas13d bears little overall similarity to other type VI effectors and shows only distant relation to Cas13a.^[^ ^39^, ^40^ ^]^ Nevertheless, its ternary structure bears close resemblance to Cas13a effectors (Figures 2A,B and 4A,B). Most of the type VI‐D CRISPR‐Cas loci originate from benign Gram‐positive gut bacteria of the genera *Ruminococcus* and *Eubacterium*.^[^ ^39^, ^40^ ^]^ Similarly to type VI‐A and type VI‐B loci, type VI‐D loci are specific for, with few exceptions, notable divergence of locus arrangement and the lack of the Cas1–Cas2 adaptation module in their relative vicinity (Figure 1A).^[^ ^39^, ^40^ ^]^ The crRNA repeat regions are highly conserved in predicted length and secondary structure, with 30 nt length, an 8–10 nt long stem with A/U‐rich loop, and a 5′‐AAAAC motif at the 3′ terminus (**Figure** **4**C).^[^ ^39^ ^]^ In line with other type VI systems, analysis of type VI‐D spacers revealed that many of these sequences target DNA phage genomic sequences and not RNA phages as previously suggested.^[^ ^40^, ^65^ ^]^

Cas13d processes the associated CRISPR array into mature crRNAs with 5′ 30 nt repeat region.^[^ ^39^ ^]^ In vitro pre‐crRNA processing experiments indicate that the 3′ direct repeat region is not entirely processed by Cas13d, leaving a 6 nt truncation that remains an extension to spacer and is randomly truncated in vivo by other ribonucleases to yield mature crRNAs with spacers of varying lengths.^[^ ^40^ ^]^ On the whole, pre‐crRNA processing by Cas13d is speculated to adopt a base‐catalyzed mechanism in which highly conserved basic residues from HEPN‐2 domain play a crucial role.^[^ ^66^ ^]^ The presence of divalent metal ions is not required for pre‐crRNA processing, but may increase processing efficiency at lower Cas13d‐crRNA ratios by increasing the binding affinity of Cas13d for crRNA.^[^ ^66^, ^67^ ^]^

Like other Cas13 effectors, the bipartite HEPN motif‐mediated RNase activity of Cas13d relies on the presence of spacer‐complementary activator RNA and Mg^2+^ ions.^[^ ^39^, ^40^ ^]^ Cas13d cleaves ssRNA sequences with preference for uracil bases and minimal secondary structure, and no PFS‐imposed constraints.^[^ ^39^, ^40^, ^67^ ^]^ Although Cas13d is also reported to exhibit robust collateral nonspecific RNase activity in vitro, such activity was not observed in mammalian cells^.[^ ^39^ ^]^ Importantly, robust cleavage activity of Cas13d is maintained through a wide temperature range (24–41 °C), which makes it useful for application in broad range of hosts.^[^ ^39^ ^]^ Moreover, most of the VI‐D loci have an adjacent WYL domain‐containing accessory protein that augments ssRNA cleavage in a dose‐dependent manner.^[^ ^40^, ^68^ ^]^ The reported structure of *Ruminococcus* sp. WYL1 (RspWYL1) accessory protein provides insights into mechanisms underlying specific binding of ssRNA substrates by WYL1 and modulation of Cas13d activity.^[^ ^68^ ^]^

### 4.1 Domain Organization

Recently published apo, binary, and ternary cryo‐EM structures of *Eubacterium siraeum* Cas13d (EsCas13d) and high‐resolution binary structure of uncultured *Ruminococcus* sp. Cas13d (UrCas13d or RspCas13d) show that Cas13d roughly adopts bilobed architecture reminiscent of larger class 2 effectors (Figure 4A,B).^[^ ^66^, ^67^ ^]^ The protein contains five domains divided into an REC lobe and an NUC lobe: NTD and Helical‐1 domains form the REC lobe, whereas the NUC lobe comprises HEPN‐1, Helical‐1, Helical‐2, and HEPN‐2 domains. HEPN‐1 domain provides a structural scaffold interconnecting the two lobes and acts as a hinge (Figure 4A,B).^[^ ^66^, ^67^ ^]^ In terms of primary sequence, Cas13d has counterparts to every domain of Cas13a except the Helical‐1 domain of Cas13a.^[^ ^40^ ^]^ Due to its compact size, all five domains are essential for RNase activity of Cas13d, and only partial truncations of the Helical‐2 domain were tolerated in EsCas13d.^[^ ^67^ ^]^

### 4.2 Binary Complex and crRNA Recognition

Binding to cognate crRNA stabilizes the protein structure, particularly the dynamic REC lobe and parts of HEPN‐2 domain, and results in formation of a positively charged solvent‐exposed channel between REC and NUC lobes.^[^ ^67^ ^]^ In this channel, the crRNA repeat region is clamped between NTD, HEPN‐1, and HEPN‐2 domains and spacer region is sandwiched within a channel formed by all domains except NTD (Figure 4A,B).^[^ ^66^, ^67^ ^]^ Because of compact size of Cas13d, a part of stem‐loop of the repeat region protrudes into solvent, which allows rational engineering of crRNA by truncation of redundant nucleotides within this region.^[^ ^66^ ^]^ Extensive interactions with both sugar‐phosphate backbone and nucleobases are formed between Cas13d and crRNA repeat region.^[^ ^66^, ^67^ ^]^ Most of these interactions are concentrated within the 3′‐end of the crRNA repeat region, where conserved base‐specific contacts play an important role in maintaining proper crRNA binding and positioning.^[^ ^66^, ^67^ ^]^ Both structure and sequence of crRNA repeat region are essential for ssRNA cleavage.^[^ ^66^ ^]^ Notably, two hydrated Mg^2+^ ions were found to be important for conformational stabilization of the UrCas13d crRNA repeat region: (1) a pentahydrated Mg^2+^ ion located at the center of the U‐shaped turn formed by the 3′ nucleotides of the crRNA repeat region (Figure 4D) and (2) a tetra‐hydrated Mg^2+^ ion that aids in stabilization of the crRNA repeat region‐interacting loop in NTD domain of Cas13d, thus indirectly contributing interactions for further conformational stabilization of the crRNA repeat region.^[^ ^66^ ^]^

In the binary complex, the crRNA spacer region adopts a conformation with three U‐shaped turns.^[^ ^66^, ^67^ ^]^ Interaction between Cas13d and spacer region is maintained mostly through sugar‐phosphate backbone and is responsible for stabilizing the spacer conformation and arranging it for activator RNA binding.^[^ ^66^, ^67^ ^]^

### 4.3 Ternary Complex and Activator RNA Binding

Upon activator RNA binding, the spacer abolishes most of its previous interactions with Cas13d and reorganizes to form a double‐stranded A‐form RNA helix with activator RNA.^[^ ^67^ ^]^ Concurrently, new interactions between Cas13d (NTD, HEPN‐1, Helical‐1, and Helical‐2 domains) and phosphate backbones of crRNA spacer and activator RNA are formed. Mutating conserved Cas13d residues that contact crRNA and activator RNA abolishes ssRNA cleavage, implying importance of Cas13d‐mediated stabilization of the crRNA‐activator RNA duplex for HEPN nuclease site activation.^[^ ^67^ ^]^

During transition from binary to ternary complex, Cas13d undergoes numerous conformational changes to accommodate the target RNA in the positively charged channel and reconfigure the bipartite HEPN nuclease active for cleavage of ssRNA.^[^ ^67^ ^]^ Activation of the HEPN nuclease active site is strongly interlinked with activator RNA binding, with minimum of 18‐nucleotide strict complementarity required for partial conformational rearrangement of Cas13d, 18–20‐nucleotide complementarity for half‐maximal cleavage activity, and >21‐nucleotide complementarity for optimal cleavage activity (i.e., full conformational activation of Cas13d).^[^ ^39^, ^67^ ^]^ The Cas13d crRNA spacer was initially thought to lack a clear seed region.^[^ ^67^ ^]^ However, studies on UrCas13d suggest that two distinct regions of UrCas13d crRNA spacer, namely the internal region (spacer nucleotides 5–8) and 3′‐end region (spacer nucleotides 13–22), are intolerant to mismatches with activator ssRNA and abolish ssRNA cleavage (Figure 4E).^[^ ^66^ ^]^ More recently, *Ruminococcus flavefaciens* XPD3002 Cas13d (RfxCas13d, CasRx) was systematically tested for mismatch intolerance, and the spacer region between nucleotides 15–21 with center at nucleotide 18 was found to be most susceptible to mismatches, whereas nucleotides of the internal region were more tolerant to mismatches.^[^ ^69^ ^]^ Thus, currently available data indicates the presence of a highly mismatch‐intolerant region within central–3′ spacer nucleotides and an internal region whose mismatch tolerance presumably varies in ortholog‐dependent manner.

## 5 Applications of Type VI CRISPR‐Cas Systems

In the years following their discovery, biochemical and structural characterization, type VI CRISPR‐Cas systems have attracted much attention because of efficient, highly specific, and programmable RNA‐targeting properties and autonomous pre‐crRNA processing. Transcriptome editing by Cas13 effectors also has important advantages over CRISPR‐based genome editing: it is safer because of its transient and reversible nature, and the extent of editing is dose‐dependent and can be modulated to suit various purposes. In addition, type VI CRISPR‐Cas systems exhibit collateral ssRNA cleavage activity in bacteria and in vitro, which can be utilized for diagnostic applications. Thus, an increasing number of studies have aimed to develop type VI CRISPR‐Cas systems into tools for basic research, biotechnology, and therapeutics. Most of these studies have recently been extensively reviewed and compared to other RNA‐targeting tools elsewhere.^[^ ^24^, ^33^, ^35^, ^36^, ^70^, ^71^, ^72^, ^73^, ^74^, ^75^, ^76^, ^77^, ^78^, ^79^ ^]^ This article will give an overview of the reported applications, their principles and drawbacks before discussing possible strategies for optimizing efficiency and safety of the Cas13‐based tools.

### 5.1 Transcriptome Engineering in Basic Research and Therapeutics

**Figure 5:**
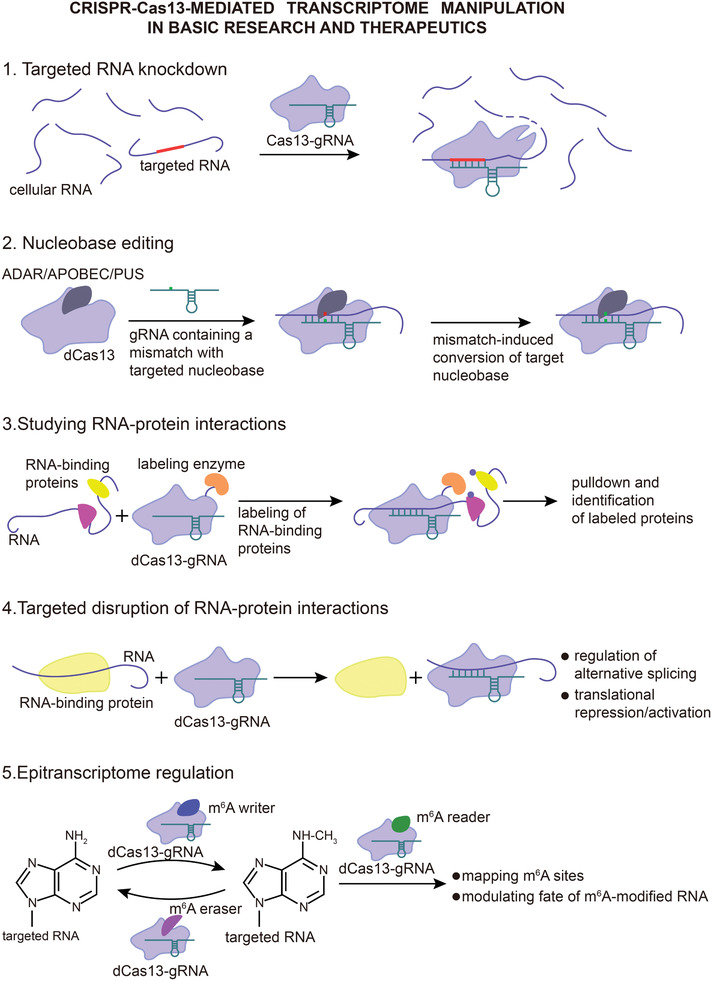
Applications of type VI CRISPR‐Cas systems in transcriptome engineering. Type VI CRISPR‐Cas systems can be used to manipulate cellular RNA in numerous ways both for basic research and therapeutics. While catalytically active Cas13 variants can be applied in targeted RNA knockdown, the fact that inactive Cas13‐gRNA complex retains specific RNA‐binding properties allows targeted disruption of RNA binding by other proteins in applications such as regulation of alternative splicing. Fusing RNA‐modifying enzymes to Cas13 effectors further expands transcriptome manipulation methods to site‐directed nucleobase editing, epitranscriptome modulation (e.g., regulation of m^6^A modifications) and labeling of RNA‐interacting proteins. Other RNA manipulation methods such as translational activation/repression and localization may be achieved by linking inactive Cas13 effectors to certain signal peptides. Abbreviations: ADAR, adenosine deaminase acting on RNA; APOBEC, apolipoprotein B mRNA editing enzyme, catalytic polypeptide‐like; PUS, pseudouridine synthase.

Cellular RNA, both protein‐coding and noncoding, is fundamentally involved in a plethora of biological processes, including conveying genetic information for protein synthesis and playing diverse regulatory roles by interacting with proteins, DNA and other RNA molecules. Conversely, the fate of cellular RNA is determined by similarly diverse protein‐ and RNA‐mediated regulation and modifications. While substantial advances in our understanding of these processes have been made through genome‐scale observational studies, their functional characterization has been largely obstructed due to limited precision, efficiency, and utility of the commonly used tools for RNA manipulation such as RNAi and antisense nucleotides.^[^ ^35^ ^]^ Recent studies, however, show that type VI CRISPR‐Cas systems can overcome these barriers and provide new strategies for RNA manipulation (**Figure** **5**). Additionally, Cas13‐based transcriptome engineering for therapeutic purposes holds promise as safer and more versatile approach compared to the DNA‐targeting CRISPR‐Cas systems.

In one of the earliest reports on Cas13 application, Cox et al. linked C‐terminally truncated catalytically inactive *Prevotella* sp. P5‐125 Cas13b (dPspCas13b) to the hyperactive mutant of deaminase domain of ADAR2 (adenosine deaminase acting on RNA 2) to develop a tool termed REPAIRv2 (RNA editing for programmable A to I replacement version 2) that can be packaged into adeno‐associated viral (AAV) vector for cell delivery.^[^ ^42^ ^]^ Accompanied with crRNA sequence targeting the gene of interest, REPAIRv2 conducts reversible and directed adenosine‐to‐inosine (A‐to‐I) edits on RNA transcripts; since inosine mimics guanosine in translation and splicing, REPAIRv2 can be used to study or treat disease‐relevant G to A mutations.^[^ ^42^ ^]^ Further engineering of REPAIRv2 aimed at relaxing substrate preferences of ADAR2 deaminase domain yielded a new tool termed RESCUE (RNA editing for specific C to U exchange), which is capable of carrying out cytidine‐to‐uridine (C‐to‐U) conversions while retaining the original adenosine deaminase activity.^[^ ^80^ ^]^ Although both REPAIRv2 and RESCUE exhibit high specificity (20 or less transcriptome‐wide off‐target edits detected upon transfection of 10 ng of REPAIRv2 vector, and 103 C‐to‐U and 139 A‐to‐I transcriptome‐wide off target edits detected upon transfection of 150 ng of RESCUE vector), their current efficiency (up to 30% of on‐target A‐to‐I edits by REPAIRv2 and ≈76% of on‐target C‐to‐U edits by RESCUE) still has room for improvement.^[^ ^42^, ^80^ ^]^

Xu et al. established the first high‐throughput phenotypic assay for functional studies of long noncoding (lnc) RNAs using *Leptotrichia wadei* Cas13a (LwaCas13a), which solves the problems observed with methods based on RNAi or antisense nucleotides, such as poor specificity, off‐target effects, and ambiguity in assigning phenotypes to a single lncRNA.^[^ ^81^ ^]^ In the assay, a library of K562 chronic myeloid leukemia cells stably expressing LwaCas13a and a unique crRNA targeting one of lncRNA transcripts was exposed to cancer drug‐induced cellular stress, after which the roles of the studied lncRNAs in K562 cell viability were inferred from depletion or enrichment of each crRNA in the assayed cell population.^[^ ^81^ ^]^

Mapping RNA–protein interactions is essential for better understanding of various cellular processes.^[^ ^82^ ^]^ Although efficient and robust methods such as cross‐linking immunoprecipitation sequencing and RNA immunoprecipitation sequencing are broadly applied to identify RNAs bound to proteins of interest, currently available methods for detection of proteins bound to a specific RNA have severe limitations, including nonspecific binding and inability to detect weaker or transient interactions.^[^ ^83^, ^84^ ^]^ To overcome these limitations, two Cas13‐based methods have recently been developed for the RNA‐centered study of RNA–protein interactions under natural conditions.^[^ ^83^, ^84^ ^]^ The first method, termed CRUIS (CRISPR‐based RNA‐united interacting system), employs catalytically inactivated LwaCas13a (dLwaCas13a)‐crRNA module to bind the RNA of interest, after which the bacterial ligase proteasomal accessory factor A (PafA) fused to dLwaCas13a ligates the small protein prokaryotic ubiquitin‐like protein PupE to RNA‐bound neighboring proteins.^[^ ^83^ ^]^ The second method uses dRfxCas13d fused with (1) the double‐stranded RNA‐binding domain from human protein kinase R that would enhance and stabilize binding to targeted RNA after initial recognition by the dRfxCas13d‐gRNA and (2) the modified plant peroxidase APEX2 which catalyzes 1 min promiscuous biotinylation of transiently interacting proteins for proximity labeling.^[^ ^84^ ^]^ The two methods were successfully used to identify new endogenous interacting partners of the noncoding RNA activated by DNA damage (NORAD) and human telomerase RNA, respectively.^[^ ^83^, ^84^ ^]^ It should be noted that careful design and screening of gRNAs is required for these two methods to find appropriate targeting sites within RNA of interest, as secondary RNA structure and possible steric hindrance caused by the size of Cas13 effectors may negatively affect target RNA binding and/or detection of some interacting proteins.^[^ ^83^, ^84^ ^]^

Type VI CRISPR‐Cas systems also have considerable potential for implementation in research on various model organisms. Aiming to establish a cost‐efficient and robust technique that would circumvent cytotoxic and off‐target effects of the commonly used morpholinos, Kushawah et al. recently assessed the utility of type VI CRISPR‐Cas systems in studying gene function in early development of teleost embryos.^[^ ^85^ ^]^ Among investigated Cas13 effectors, RfxCas13d was found to efficiently and precisely disrupt maternal and zygotic gene function in zebrafish embryos without inducing toxicity, developmental abnormalities, direct off‐target or collateral cleavage effects.^[^ ^85^ ^]^ The study also demonstrated that coinjection of RfxCas13d protein and gRNAs can further accelerate knockdown of targeted maternal mRNA in dose‐dependent manner and provide more penetrant phenotypes.^[^ ^85^ ^]^ The established technique can be effectively used in other vertebrate embryos, including those of medaka, killifish, and mouse.^[^ ^85^ ^]^ In another study related to model organisms, Jing et al. showed that type VI CRISPR‐Cas systems can be used for RNA knockdown and single‐base RNA editing in the fission yeast *Schizosaccharomyces pombe*, an important model organism used for studying cellular mechanisms conserved from yeast to humans.^[^ ^86^ ^]^

To investigate one potential therapeutic application, Konermann et al. delivered dRfxCas13d to cells via AAV vector to manipulate pathological alternative splicing of tau pre‐mRNA in a neuronal model of frontotemporal dementia.^[^ ^39^ ^]^ Later, Zhao et al. showed that Cas13 effectors can also be considered for therapeutic knockdown of oncogenes for which the use of small molecule inhibitors has thus far proven unsuccessful.^[^ ^45^ ^]^ Programmed by crRNA to specifically target oncogenic kirsten rat sarcoma virus (KRAS) mutant and not wild‐type KRAS mRNA transcripts, LwaCas13a efficiently decreased levels of mutant KRAS mRNA, inducing cell apoptosis in pancreatic cancer cells and tumor shrinkage in mice with pancreatic cell xenografts.^[^ ^45^ ^]^ Furthermore, He et al. delivered active RfxCas13d to mouse liver for simultaneous and reversible knockdown of RNA transcripts associated with metabolic regulation, thus laying the groundwork for the use of type VI CRISPR‐Cas systems in treatment of metabolic diseases.^[^ ^87^ ^]^

In addition to transcriptome manipulation, Cas13‐based tools can also be used for studying and therapeutic regulation of epitranscriptome. One of the most prominent and abundant mRNA modifications is the N^6^‐methyladenosine (m^6^A), a form of reversible and selective posttranscriptional methylation of adenosine residues that influences alternative splicing, conformation, expression, translation, and degradation of mRNA transcripts.^[^ ^88^, ^89^ ^]^ The effects of m^6^A modifications on mRNA are mediated by m^6^A reader, writer, and eraser proteins.^[^ ^88^, ^89^ ^]^ Previous transcriptome‐wide m^6^A mappings suggest that distribution and pattern of m^6^A modifications are dynamic and associated with cell differentiation and various diseases such as cancers; however, the lack of appropriate tools and methods has hitherto hindered deeper understanding of involvement of m^6^A‐mediated epitranscriptome regulation in cellular processes and diseases.^[^ ^88^, ^89^ ^]^ To study m^6^A regulation on individual RNA transcripts, Rauch and Dickinson recently designed a protocol in which dCas13b effectors fused to the functional output domains of the m^6^A reader proteins YTH domain‐containing family protein 1 and 2 (YTHDF1 and YTHDF2) were transfected to cells along with crRNA targeting methylated sites in RNA transcript of interest, followed by assessment of protein levels with dual luciferase assay or RT‐qPCR for evaluation of RNA levels.^[^ ^90^ ^]^ In another study, the m^6^A eraser protein RNA demethylase alpha‐ketoglutarate‐dependent dioxygenase homolog 5 (ALKBH5) was linked to the C‐terminus of dPspCas13b.^[^ ^91^ ^]^ When combined with gRNA, the dPspCas13b‐ALKBH5 fusion protein (named dm^6^ACRISPR) successfully demethylated targeted mRNA transcripts containing single or multiple m^6^A sites in human cell culture with low off‐target effects.^[^ ^91^ ^]^ dm^6^ACRISPR was also used to target m^6^A‐modified transcripts of the oncogenes EGFR and MYC, which suppressed proliferation of HeLa cells and demonstrated that dm^6^ACRISPR can be applied in gene repression and regulation of cellular functions.^[^ ^91^ ^]^ In the third study, Zhao et al. developed a photoactivable RNA m^6^A editing system using CRISPR‐dCas13 (PAMEC) that includes two main components: (1) an RNA anchor probe consisting of the catalytically inactive *Porhyromonas gulae* Cas13b (dPguCas13b) fused to CIBN, a truncated variant of the light‐sensitive protein calcium‐ and integrin‐binding 1 (CIB1) that mediates light‐dependent interaction with the photylase homology region of the cryptochrome circadian regulator (CRY2PHR), and (2) an m^6^A effector probe consisting of CRY2PHR fused to either m^6^A demethylase fat‐mass and obesity‐associated protein (FTO) for m^6^A erasure or the METTL3‐METTL14 m^6^A methyltransferase complex for m^6^A writing.^[^ ^92^ ^]^ The system enables efficient and robust m^6^A editing in cells illuminated by blue light, and it has been further optimized by adding an MS2 aptamer at 3′ end of crRNA for increased efficiency and by coupling it with an upconversion nanoparticle film for deep tissue m^6^A editing.^[^ ^92^ ^]^ Considering significant attention that has lately been given to research on m^6^A‐mediated epitranscriptome regulation and promising prospects of Cas13 effectors, it is likely that even more Cas13‐based tools fused to various m^6^A reader/writer/eraser proteins will be developed in the near future.

### 5.2 Nucleic Acid Detection and Diagnostics

**Figure 6:**
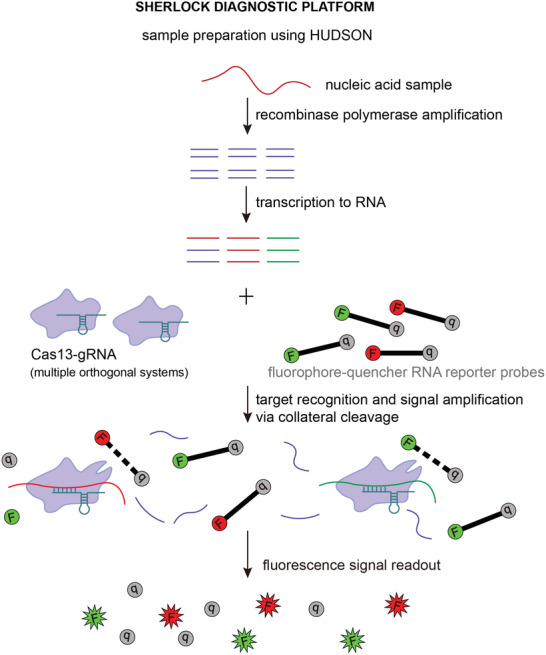
Schematic representation of Cas13‐based nucleic acid detection using SHERLOCK (specific high‐sensitivity enzymatic reporter unlocking) diagnostic platform. SHERLOCK harnesses the nonspecific ssRNA cleavage activity of Cas13 effectors exhibited after detecting the guide RNA‐complementary RNA sequence to determine the presence and quantify the amount of certain nucleic acid species in a sample. Briefly, collected samples are first treated using HUDSON (heating unextracted diagnostic samples to obliterate nucleases), after which nucleic acid samples are converted to cDNA via recombinase polymerase amplification and then reverted to RNA by T7 transcription. The sample is then mixed with short RNA reporter fragments (e.g., poly‐U or poly‐A sequences) containing a fluorophore‐quencher pair. Incubation with Cas13 and target‐specific guide RNA initiates Cas13‐mediated nonspecific ssRNA cleavage, including cleavage of ssRNA reporters that emit fluorescent light proportionally to the amount of targeted nucleic acid species, which enables visualization and quantitation of results via fluorescence or colorimetric lateral flow readout. SHERLOCK can be used for diagnosing viral and other infectious diseases, cancer mutations, health‐related single nucleotide polymorphisms, genetic traits in plants, etc.

By harnessing the collateral cleavage activity and stringent crRNA‐activator RNA complementarity requirements of type VI CRISPR‐Cas systems, Gootenberg et al. designed a rapid, cheap, and ultrasensitive portable nucleic acid diagnostic platform named SHERLOCK (specific high‐sensitivity enzymatic reporter unlocking).^[^ ^43^ ^]^ SHERLOCK uses a Cas13‐crRNA module that, once activated by a strictly complementary activator RNA from tested sample, promiscuously cleaves quenched fluorescent reporter RNA molecules at high turnover rate, thus generating a detectable and quantitative fluorescent signal (**Figure** **6**).^[^ ^43^ ^]^ The preceding recombinase polymerase amplification of samples followed by T7 transcription enables detection of both RNA and DNA samples at zeptomolar level.^[^ ^43^, ^44^ ^]^ Subsequent development and standardization of the platform allowed detection of multiple target molecules using orthogonal type VI CRISPR‐Cas systems, enhanced signal output by adding the type III CRISPR effector nuclease Csm6, simplified sample preparation by introducing the method termed HUDSON (heating unextracted diagnostic samples to obliterate nucleases) and adapted the platform for convenient visual readout of results on a lateral flow strip.^[^ ^44^, ^93^, ^94^ ^]^ SHERLOCK has been applied in diagnostics of viral infections, including COVID‐19, cancer mutations, health‐related single nucleotide polymorphisms (SNPs), and genetic traits in plants.^[^ ^43^, ^44^, ^94^, ^95^, ^96^, ^97^, ^98^, ^99^, ^100^ ^]^ The utility of SHERLOCK as a cheap and reliable tool for diagnosing COVID‐19 was recently evaluated on a larger scale in a clinical study conducted in Thailand.^[^ ^101^ ^]^ Validated on 154 clinical COVID‐19 samples, SHERLOCK exhibited 100% specificity and high sensitivity with both in‐tube fluorescence and lateral flow readouts (96% and 88% sensitivity, respectively), successfully detected asymptomatic cases and its results were in full concordance with those from the commonly used RT‐PCR tests.^[^ ^101^ ^]^ SHERLOCK has also been adapted for simple, fast, and cheap monitoring of graft rejection and opportunistic infections by cytomegalovirus and BK polyomavirus in kidney transplant patients.^[^ ^102^ ^]^ Available in form of qualitative lateral‐flow readout that can be evaluated by a smartphone‐based software, the test enables frequent and personalized point‐of‐care testing for early prevention of post‐transplantation complications.^[^ ^102^ ^]^

As the COVID‐19 pandemic highlighted the need for faster and less labor‐intensive diagnostic tools with minimal equipment usage, SHERLOCK was recently further simplified into the diagnostic platform termed SHINE (SHERLOCK and HUDSON integration to navigate epidemics).^[^ ^95^ ^]^ SHINE uses optimized protocol for HUDSON to speed up viral inactivation in nasopharyngeal swabs and saliva samples and combines the recombinase polymerase amplification, T7 transcription, and Cas13‐mediated detection steps of SHERLOCK into a single‐step reaction, thereby reducing time for obtaining COVID‐19 test results to ≈50 min while maintaining 100% specificity and 90% sensitivity compared to RT‐PCR tests.^[^ ^95^ ^]^ In addition to lateral flow readout, SHINE test results can be visualized via in‐tube fluorescent readout and interpreted by a smartphone application to avoid sample contamination and user bias.^[^ ^95^ ^]^

Apart from SHERLOCK, other Cas13‐based diagnostic tools have been developed for various purposes, such as quantitative detection of microRNA or quantitative virus detection using automated microfluidic device.^[^ ^48^, ^103^, ^104^ ^]^ Among these tools, CARMEN (combinatorial arrayed reactions for multiplexed evaluation of nucleic acids) should be noted for its capability to perform large‐scale simultaneous testing of multiple samples for diverse pathogens at species, strain and SNP level—a feature particularly valuable for surveillance of spreading and evolution of infectious diseases.^[^ ^104^ ^]^

### 5.3 Nucleic Acid Imaging

**Figure 7:**
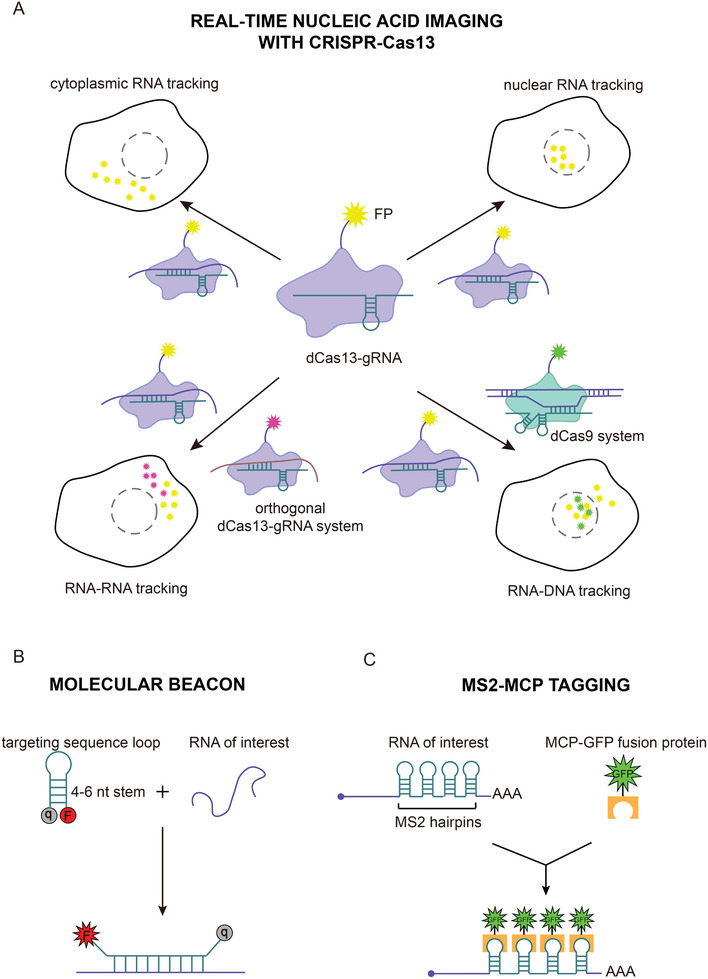
Real‐time nucleic acid imaging and tracking in living cells using type VI CRISPR‐Cas systems. A) Fusing a fluorescent protein (FP) to a catalytically inactive Cas13 effector and coupling it with guide RNA specific for the RNA of interest can be used in spatiotemporal visualization of the targeted RNA transcripts in living cells. Further addition of an orthogonal Cas13 system or a DNA‐targeting Cas9 system fused to a different fluorophore enables dual RNA–RNA or RNA–DNA imaging, respectively. B,C) Molecular beacons and MS2‐MCP (MS2‐coating protein) tagging are two commonly used tools for real‐time RNA tracking in live cells. B) Molecular beacons are 20–25 nt oligonucleotide probes held in a stem‐loop conformation. One end of the probe contains a fluorophore molecule whose fluorescent light emission is inhibited by a quencher molecule at the other end of the probe. The stem‐loop structure dissociates when the loop containing the targeting sequence hybridizes with RNA of interest, which separates fluorophore from quencher and thereby allows emission of detectable fluorescent light. C) MS2‐MCP RNA tagging requires transfection of two plasmids into host cell: one plasmid encoding RNA interest with multiple MS2 hairpin sequences cloned downstream (often within 3′ untranslated region), and another plasmid encoding MCP fused to green fluorescent protein (GFP). The MCP–GFP fusion proteins bind to MS2 hairpins within the ectopically expressed RNA of interest, emitting fluorescent signal proportional to the number of MS2 hairpins.

Subcellular localization of RNA transcripts is spatiotemporally dynamic and directly associated with their function and fate.^[^ ^78^ ^]^ Although fluorescent tools such as molecular beacons, the MS2‐MCP system and fluorogenic RNA aptamers are available, their limitations make tracking individual RNA species in live cells difficult (**Figure** **7**A–C).^[^ ^78^, ^105^ ^]^ Wang and co‐workers demonstrated that catalytically inactive dPspCas13b and dPguCas13b fused to enhanced green fluorescent protein can be employed as robust and fast RNA‐labeling tools for real‐time RNA imaging and tracking in living cells, with signal‐to‐noise ratio lower than that of MS2‐MCP systems.^[^ ^106^ ^]^ Both Cas13b orthologs were capable of efficient binding to cellular RNAs with medium level of abundance, and could be combined with an orthogonal dCas13 or MS2‐MCP system for dual‐color RNA–RNA imaging, or with a dCas9 system for RNA–DNA imaging (Figure 7A).^[^ ^106^ ^]^ More recently, the same research group also published a detailed protocol for the aforementioned dCas13b‐mediated imaging of RNA in living cells.^[^ ^107^ ^]^ The protocol thoroughly describes every step, including gRNA design, selection of fluorescent proteins, preparation of cells for microscopy, data analysis, and troubleshooting.^[^ ^107^ ^]^ Moreover, inactive RfxCas13d coupled with fluorescent‐labeled crRNA has been used along with a dCas9‐fluorescent crRNA system for real‐time simultaneous visualization of transcript RNA and genomic DNA in the method known as CRISPR LiveFISH (live‐cell fluorescent in situ hybridization).^[^ ^108^ ^]^

### 5.4 Antiviral Applications

**Figure 8:**
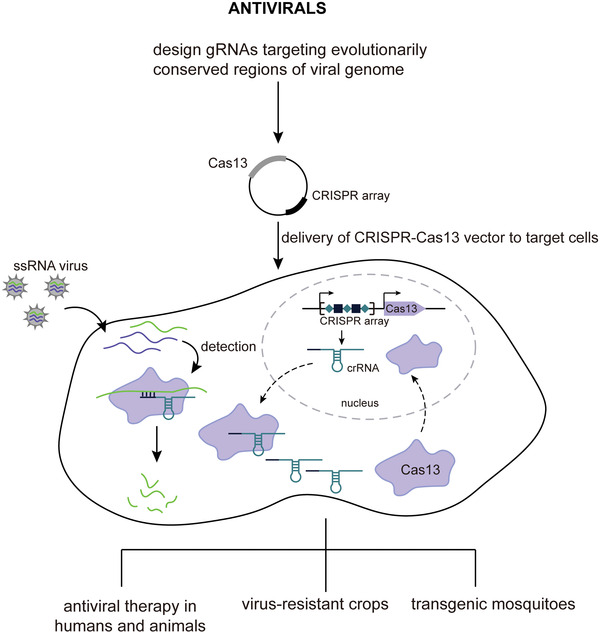
Antiviral applications of type VI CRISPR‐Cas systems. Programmed to target evolutionarily conserved genomic regions of ssRNA viruses or RNA intermediates of DNA viruses, type VI CRISPR‐Cas systems can be used to suppress viral infections in humans and animals, or to develop virus‐resistant crops and transgenic mosquitoes with reduced capacity for spreading viral diseases.

Vaccines and commonly used antivirals (i.e., small‐molecule inhibitors and monoclonal antibodies) are often hindered by costliness, long development time, and viral resistance that arises from high mutation rates.^[^ ^109^ ^]^ Considering the importance of viral genome replication for completion of viral life cycle in host cells, viral inhibition through CRISPR‐Cas‐mediated degradation of highly conserved and essential genetic elements could potentially be used as cheaper and more efficient antiviral strategy. The efficacy and feasibility of this strategy was initially explored with CRISPR‐Cas9 systems, which hold considerable promise in curing chronic viral infections caused by viruses with characteristic latency state such as human immunodeficiency virus, hepatitis B virus, herpes viruses and human papillomavirus.^[^ ^110^ ^]^ However, ≈51%, 44%, and 70% of genera infecting humans, vertebrate animals, and plants, respectively, are ssRNA viruses that do not use DNA intermediates during their life cycles.^[^ ^109^, ^111^, ^112^ ^]^ Even though the RNA‐targeting CRISPR‐Cas9 systems can be used, the exclusively ssRNA‐targeting type VI CRISPR‐Cas systems are more suitable for targeting ssRNA viruses (**Figure** **8**).

The potential use of Cas13 systems in protecting plants against viruses was first demonstrated with LshCas13a, which was transgenically expressed in monocot and dicot plants to interfere with replication of economically important RNA viruses; LshCas13a maintained its pre‐crRNA processing activity in plants and did not cause cytotoxic effects.^[^ ^47^, ^113^, ^114^ ^]^ RfxCas13d also exhibits robust and specific antiviral activity against RNA viruses in plants, at which it outperformed LwaCas13a and PspCas13b in both transient and stable overexpression assays; in the study, RfxCas13d did not exert collateral cleavage effect in plants and was able to efficiently target two RNA viruses in parallel when crRNAs targeting both viruses were expressed in tested plants.^[^ ^111^ ^]^ Recently published protocol describing transient expression of LbuCas13a and cognate CRISPR array in *Nicotiana benthamiana* will allow more extensive research on potential application of type VI CRISPR‐Cas systems for antiviral defense in plants.^[^ ^115^ ^]^

Antiviral properties of Cas13 have also been tested against high doses of three distinct ssRNA viruses in mammalian cell lines lacking functional innate immune system, displaying potent viral inhibitory activity without affecting cell viability.^[^ ^97^ ^]^ The authors of the study coupled Cas13‐mediated virus targeting with SHERLOCK to create a comprehensive platform for diagnosis and treatment of viral diseases termed CARVER (Cas13‐assisted restriction of viral expression and readout).^[^ ^97^ ^]^ In the wake of COVID‐19 pandemic and demands for alternative therapeutic approaches created by the lack of effective treatments and time required for vaccine development, Abbott et al. demonstrated the potential of Cas13‐based therapy in combating emerging viral diseases.^[^ ^116^ ^]^ Selecting RfxCas13d due to absence of PFS‐imposed cleavage restrictions, robust and highly specific RNA interference and its compact size that would facilitate packaging and delivery, the authors developed PAC‐MAN (prophylactic antiviral CRISPR in human cells) strategy for inhibiting SARS‐CoV‐2 and influenza A virus, as well as the majority of other coronavirus and influenza virus strains, by simultaneously targeting multiple highly conserved viral genomic regions.^[^ ^116^ ^]^ The RfxCas13d‐based PAC‐MAN was shown to efficiently cleave SARS‐CoV‐2 RNA fragments and inhibit influenza A virus replication in human lung epithelial cell cultures, with greater inhibition at higher crRNA concentrations and lower viral titers.^[^ ^116^ ^]^ The results of the study indicated that PAC‐MAN could be effectively used for preventing new viral infections or as a complementary strategy along with traditional pharmaceuticals and vaccines.^[^ ^116^ ^]^

A variety of RNA viruses also infect domestic animals, thereby causing substantial economic losses. To investigate feasibility of repressing RNA virus infections in animals and humans with CRISPR‐Cas13b systems, Cui et al. used PspCas13b to target two essential genes of the porcine reproductive and respiratory syndrome virus in infected mammalian cells and almost completely inhibited viral gene transcription and expression.^[^ ^117^ ^]^ Taking into account that delivery of Cas effector and gRNAs/CRISPR array via separate plasmids negatively affects targeting efficiency due to inconsistent expression levels of each component, the authors of the study also developed a single‐vector delivery system that facilitates multiplexed targeting and further increases interference levels.^[^ ^117^ ^]^

In addition to potential application in targeting viruses in plants and mammals, type VI CRISPR‐Cas systems could also be used in mosquitoes for suppressing mosquito‐borne viral diseases such as dengue fever, chikungunya, and Zika. Tng et al. tested PspCas13b against a chimeric firefly luciferase reporter carrying the chikungunya virus (CHIKV) genomic sequence corresponding to the region encoding the nonstructural protein 2 (nsP2) and a CHIKV split replication system mimicking replication of viral RNA.^[^ ^118^ ^]^ Guided by two gRNAs targeting nsP2 or CRISPR array targeting multiple sites in viral genome, PspCas13b performed efficiently against both the chimeric luciferase reporter and the CHIKV split replication system and significantly reduced viral RNA expression.^[^ ^118^ ^]^ Interestingly, the two gRNAs targeting nsP2 were also capable of knocking down viral RNA in the absence of PspCas13b; further investigation indicated that addition of PspCas13b enhanced viral RNA knockdown when U6 promoter‐driven guides were used but did not increase suppression in cases when in vitro transcribed gRNAs were directly delivered to cells.^[^ ^118^ ^]^ Since this phenomenon has not been observed in mammalian and plant cells, it is likely specific for mosquito cells, possibly because insect cells are generally more reliant on endogenous RNA interference systems that could utilize gRNAs to target viral sequences independently of PspCas13b.^[^ ^118^ ^]^

## 6 Future Perspectives

Although previous structural and functional studies provided a firm basis for leveraging type VI CRISPR‐Cas systems into RNA‐targeting tools, certain mechanisms underlying the functions of Cas13 effectors are still not fully understood, which hinders more sophisticated and standardized application. As the conclusion of this review, we discuss open questions that need to be addressed in order to improve performance and safety of Cas13‐based tools.

### 6.1 Mechanisms for HEPN Nuclease Site Activation and ssRNA Cleavage

Although fundamental insights into conformational changes through which Cas13 effectors undergo during transition from binary to ternary complex have already been provided, mechanisms underlying the binding of activator RNA, conformational activation of the HEPN nuclease site and ssRNA cleavage are still largely unknown. Understanding which subregions of crRNA spacer play crucial roles in activator RNA binding and HEPN nuclease site activation is particularly important for designing efficient crRNAs. Tambe et al. have shown that activator RNA binding and HEPN nuclease site activation in LbuCas13a are governed by two separate spacer subregions, i.e., the central seed region and the “nuclease switch” region.^[^ ^56^ ^]^ This information carries implications for different approaches to crRNA guide design depending on purposed application: while avoiding mismatches in the seed and “nuclease switch” regions would be necessary when designing crRNA guides for RNA interference, introducing mismatches within the “nuclease switch” region of crRNA guides would be plausible to promote tighter binding in applications that use inactive Cas13 variants, such as RNA imaging, labeling of RNA‐interacting proteins, epitranscriptome regulation, etc. However, it is unclear to what extent the findings for LbuCas13a are applicable to other Cas13 effectors.

Elucidating the mechanisms by which ssRNAs are guided to HEPN nuclease active sites and cleaved by the catalytic residues would provide rationale for engineering Cas13‐based tools with improved cleavage efficiencies. Although more studies are necessary to validate key residues and structural elements involved in interaction with and cleavage of ssRNA, currently available data suggests that some Cas13a and Cas13b effectors possess a protruded *β*‐hairpin located proximally to the HEPN nuclease active site (Figure 2F).^[^ ^53^, ^64^ ^]^ As mentioned previously in this article, the active site‐proximal *β*‐hairpin was shown to capture RNA molecules in LbuCas13a (Figure 2F), and its truncation or deletion reduced ssRNA cleavage.^[^ ^53^ ^]^ Moreover, Slaymaker et al. have reported that deleting the active site‐proximal *β*‐hairpin in PbuCas13b (residues 938–951) abolished ssRNA cleavage activity, whereas substituting the hairpin with those from other Cas13b orthologs altered nucleotide cleavage preferences and cleavage efficiency.^[^ ^64^ ^]^ Thus, it is possible that analogous active site‐proximal structural elements in other Cas13 effectors are of similar importance. It would be worth investigating whether substitutions or modifications within such *β*‐hairpins and other active site‐proximal regions could improve RNA cleavage efficiency of Cas13 effectors for RNA knockdown and nucleic acid detection platforms such as SHERLOCK. Using Cas13 effectors with modified nucleotide cleavage preferences could further expand capacities of the nucleic acid detection platforms. In addition, verifying whether the active site‐proximal RNA‐binding regions contribute to off‐target RNA binding would be valuable for applications that make use of inactive Cas13 variants, such as REPAIRv2, RESCUE, CRUISE, and RNA imaging.

### 6.2 Optimization of crRNA Design

Aside from accurate determination of “nuclease switch” and seed regions, optimal crRNA design needs to take into account other factors, including crRNA folding (i.e., predicted secondary folding and corresponding minimum free energies), position, and accessibility of targeted RNA sequence within the transcript as well as its nucleotide composition.^[^ ^69^, ^119^ ^]^ Distribution of single‐stranded and double‐stranded regions within targeted RNA sequences also has to be considered because type VI CRISPR‐Cas systems preferably bind to and cleave single‐stranded regions, with cleavage rates significantly higher than when using crRNAs targeting double‐stranded regions.^[^ ^38^, ^40^, ^51^, ^119^ ^]^ For instance, Bandaru et al. have shown that LshCas13a‐mediated targeting of single‐stranded regions of SS18‐SSX2 transcript encoding protein promoting survival of synovial sarcoma cells led to significant decrease in cell viability compared to targeting double‐stranded regions.^[^ ^119^ ^]^ Moreover, rational design of effective and specific crRNAs based on machine‐learning techniques has already been tested on RfxCas13d—the computational Random Forest model developed by Wessels et al. was capable of distinguishing poorly performing and highly efficient RfxCas13d crRNAs, and its generalizability was confirmed by testing 3979 crRNAs on mRNA transcripts of 48 endogenous genes.^[^ ^69^ ^]^ In addition, the online tool CHOPCHOP v3 has recently incorporated options for Cas13 crRNA design based on computational predictions for secondary structures within a targeted RNA sequence and transcriptome‐level off‐target effects.^[^ ^120^ ^]^ However, crRNA design principles and machine‐learning approaches for other Cas13 effectors have not been explored yet, and transcriptome‐wide studies are required to further investigate the local secondary structure within RNA sequences and rules for design of optimal crRNA sequences.

Rational engineering of crRNA repeat region is another potential yet largely unexplored means of optimizing crRNA design and increasing RNA targeting efficiency. For example, we have shown that the crRNA stem‐loop nucleotides not bound by UrCas13d can be truncated without affecting UrCas13d cleavage activity.^[^ ^66^ ^]^ In another case, disrupting the first base pair of the guide‐proximal stem was found to notably improve RNA knockdown, possibly by stabilizing crRNA folding.^[^ ^69^ ^]^ Thus, although most alterations within crRNA repeat regions have negative impact on activities of cognate Cas proteins, structural studies and systematic screens may allow identification of certain modifications within crRNA repeat regions that could optimize crRNA design.

### 6.3 RNA Knockdown Efficiency and Its Modulation

RNA knockdown efficiency levels are known to vary among studied Cas13 orthologs, targeted RNA sequences and host cell types.^[^ ^36^, ^39^, ^41^, ^50^, ^70^ ^]^ The current lack of large‐scale systematic comparisons of Cas13 effectors with the aim of determining their RNA interference efficiencies in different host cell contexts (i.e., mammalian, plant, bacterial cells, etc.) impedes more effective use of Cas13‐based tools in RNA knockdown. Once identified, the most potent Cas13 effectors could be further enhanced by a combination of optimal crRNA design, rational crRNA, and protein engineering and use of the positively regulating accessory proteins such as Csx28 or the WYL‐domain‐containing protein to achieve near‐complete RNA knockdown levels while maintaining negligible off‐target rates. Alternatively, Cas13 effectors with lower cleavage efficiencies, suboptimal crRNA design, the negatively regulating accessory protein Csx27 or a combination of these strategies could be used for broad‐range tuning of RNA knockdown levels. In regard to accessory proteins, further studies are needed to understand how they carry out their regulatory functions and whether they could be coupled with orthogonal Cas13 effectors.

### 6.4 Temporal Regulation of Cas13 Activity

In applications such as therapeutics, RNA knockdown and binding activities of type VI CRISPR‐Cas systems need to be tightly regulated in spatiotemporal manner for targeting specific cell types, administering proper dosage and switching off the Cas13 effectors to avoid potential harmful effects. While delivery of Cas13‐based tools to target cells can be achieved using AAV vectors with specific cell tropism, Cas13 activity can be temporally regulated via chemical, radiative or enzymatic induction or via suppression with inhibitory molecules such as the accessory protein Csx27 in case of type VI‐B systems.^[^ ^70^ ^]^

Another viable strategy for regulation/inhibition of CRISPR‐Cas systems is the use of anti‐CRISPR proteins originating from bacteriophages or prophage regions of bacterial genomes.^[^ ^121^ ^]^ Several anti‐type VI‐A CRISPR proteins (AcrVIAs) were recently discovered and demonstrated to potently inhibit RNA knockdown and nucleobase editing activities of Cas13a effectors in bacteria and human cell lines without producing detrimental effects in host human cells.^[^ ^122^ ^]^ The identified AcrVIAs were able to act on multiple Cas13a orthologs, albeit more research is necessary to determine the full extent of their utility against diverse type VI‐A CRISPR‐Cas systems.^[^ ^122^ ^]^ Moreover, AcrVIA1 from the *Listeria seeligeri*‐targeting phage _Φ_LS46 has recently been functionally and structurally characterized.^[^ ^123^ ^]^ AcrVIA1 was found to bind the guide‐exposed face of the *Listeria seeligeri* Cas13a‐crRNA binary complex, interacting both with Cas13a and crRNA (predominantly the central‐3′ region of crRNA spacer) to prevent activator RNA binding and conformational activation of Cas13a.^[^ ^123^ ^]^ Despite these promising discoveries, biochemical properties of other identified AcrVIAs and diverse mechanisms by which they exert inhibitory effects need to be investigated, and potential cytotoxic and immunogenic effects should be thoroughly examined before AcrVIAs could be effectively and safely used for regulation of Cas13a activity. Moreover, since AcrVIAs inhibit exclusively Cas13a effectors,^[^ ^122^, ^123^ ^]^ anti‐type VI‐B and VI‐D CRISPR proteins remain to be identified and characterized.

### 6.5 Collateral Cleavage, Off‐Target Activity, Cytotoxicity, and Immunogenicity

Thus far, studies using type VI CRISPR‐Cas systems for RNA knockdown in eukaryotic cells have reported high targeting specificity, negligible off‐target activity and absence of collateral cleavage; similarly, no effects on cell viability have been observed.^[^ ^36^, ^39^, ^41^, ^50^, ^70^, ^97^, ^111^, ^113^, ^116^ ^]^ However, potential cytotoxic effects have not been systematically assessed, especially for the long‐term use of Cas13 effectors. Moreover, off‐targeting activity can be further reduced by careful gRNA design and rational engineering of Cas13 effectors. Particular attention should be given to improving the specificity of site‐directed RNA editing (e.g., the nucleobase editors REPAIRv2 and RESCUE), as fusing Cas13 effectors to editases that promiscuously bind RNA substantially increases off‐targeting events.^[^ ^124^, ^125^ ^]^ Several strategies can be employed to solve this issue, such as minimizing expression levels of the RNA editors, restricting the RNA editing to nucleus, and reducing RNA–editase interaction by introducing point mutations to the editases or through steric hindrance generated by inserting the editing enzyme into the middle of a Cas13 effector instead of linking it to one of its termini.^[^ ^125^ ^]^

The observed absence of collateral cleavage in eukaryotic cells should also be closely inspected to determine the intracellular factors that give rise to this phenomenon. There is a possibility that collateral cleavage occurs upon initial activation of the Cas13 effectors, but is silenced shortly after by certain endogenous pathways in response to increased RNA degradation. In such case, prolonged Cas13 activity may also cause cytotoxic effects through hyperactivation of the pathways that counteract collateral cleavage.

Since no studies using Cas13‐based tools in animal models have been published to date, nothing is known about potential system‐wide toxic effects. Nevertheless, expression of CRISPR‐Cas13 tools in undesired cell types could be largely mitigated via targeted delivery to specific cells or tissues, and unintended leakages would be less concerning than those of DNA‐targeting CRISPR‐Cas systems due to transient nature of RNA manipulation.^[^ ^70^ ^]^ Immunogenicity is likely to be one of the main challenges for application of Cas13‐based tools in therapeutics because sustained expression would usually be required to achieve desired phenotypic changes, which may trigger both innate and adaptive immune responses that could lead to cytotoxicity, inflammation and even fatality.^[^ ^70^ ^]^ Given that many CRISPR‐Cas systems originate from pathogenic bacteria (e.g., Cas9 systems from *Staphylococcus aureus* and *Streptococcus pyogenes*), immunogenicity could be further exacerbated by potentially pre‐existing antibodies and reactive T cells.^[^ ^126^ ^]^ Possible solutions to immunogenicity include immunosuppression, silencing of human T cell epitopes present on Cas13 effectors, use of type VI CRISPR‐Cas systems derived from benign bacteria, and circumvention of immune system with orthogonal Cas13 systems and AAV‐mediated delivery.^[^ ^127^, ^128^, ^129^ ^]^

### 6.6 PFS Constraints

PFSs have been reported for type VI‐A (the U‐cleaving subfamily) and type VI‐B CRISPR‐Cas systems.^[^ ^38^, ^51^ ^]^ Nevertheless, later studies suggested that PFS constraints may vary among orthologs and/or under different conditions, such as in cases of LwaCas13a and PspCas13b that exhibited unrestricted interference under tested conditions.^[^ ^41^, ^43^ ^]^ Moreover, due to divergent sequences and geometries of their crRNAs, the A‐cleaving Cas13a orthologs may be subjected to PFS constraints different from the U‐cleaving Cas13a effectors.^[^ ^50^, ^53^, ^54^, ^56^ ^]^ Thus, the presence and variation of PFS constraints among different Cas13a and Cas13b orthologs needs further investigation, as well as the exact role of PFS constraints. In case of U‐cleaving Cas13a effectors, previous studies indicated that a G at the position of the 3′ PFS does not negatively influence activator RNA binding, but rather appears to inhibit conformational activation of Cas13a, which is further exacerbated by extending base pairing into crRNA repeat region.^[^ ^53^, ^56^, ^130^ ^]^ Further confirmation of this notion would be valuable since PFS constraints would not need to be considered in applications where inactive variants of certain Cas13a orthologs are used.

## Conflict of Interest

The authors declare no conflict of interest.
